# CRISPRactivation-SMS, a message for PAM sequence independent gene up-regulation in *Escherichia coli*

**DOI:** 10.1093/nar/gkac804

**Published:** 2022-09-22

**Authors:** Marco Klanschnig, Monika Cserjan-Puschmann, Gerald Striedner, Reingard Grabherr

**Affiliations:** Christian Doppler Laboratory for Production of Next-Level Biopharmaceuticals in E. coli, Department of Biotechnology, University of Natural Resources and Life Sciences, Vienna, 1190 Vienna, Austria; Christian Doppler Laboratory for Production of Next-Level Biopharmaceuticals in E. coli, Department of Biotechnology, University of Natural Resources and Life Sciences, Vienna, 1190 Vienna, Austria; Christian Doppler Laboratory for Production of Next-Level Biopharmaceuticals in E. coli, Department of Biotechnology, University of Natural Resources and Life Sciences, Vienna, 1190 Vienna, Austria; Christian Doppler Laboratory for Production of Next-Level Biopharmaceuticals in E. coli, Department of Biotechnology, University of Natural Resources and Life Sciences, Vienna, 1190 Vienna, Austria

## Abstract

Governance of the endogenous gene regulatory network enables the navigation of cells towards beneficial traits for recombinant protein production. CRISPRactivation and interference provides the basis for gene expression modulation but is primarily applied in eukaryotes. Particularly the lack of wide-ranging prokaryotic CRISPRa studies might be attributed to intrinsic limitations of bacterial activators and Cas9 proteins. While bacterial activators need accurate spatial orientation and distancing towards the target promoter to be functional, Cas9-based CRISPR tools only bind sites adjacent to NGG PAM sequences. These circumstances hampered Cas9-guided activators from mediating the up-regulation of endogenous genes at precise positions in bacteria. We could overcome this limitation by combining the PAM independent Cas9 variant **S**pRY and a CRISPRa construct using phage protein **M**CP fused to transcriptional activator **S**oxS. This CRISPRa construct, referred to as **SMS**, was compared with previously reported CRISPRa constructs and showed up-regulation of a reporter gene library independent of its PAM sequence in *Escherichia coli*. We also demonstrated down-regulation and multi-gene expression control with SMS at non-NGG PAM sites. Furthermore, we successfully applied SMS to up-regulate endogenous genes, and transgenes at non-NGG PAM sites, which was impossible with the previous CRISPRa construct.

## INTRODUCTION

The overall metabolic burden increases when foreign genes are expressed in bacterial production cells, as the regulatory network becomes unbalanced (1–5). Therefore, it is key to rebalance the gene regulatory network to provide the best possible conditions for recombinant protein expression in production hosts. In *Escherichia coli*, it has been shown that co-expression of critical factors such as chaperones can unburden the metabolic load which helps to increase the product yield when folding and solubility is crucial (6–9). In contrast, down-regulation or even knockouts of proteases provides better stability of the product (4,10–12). However, the specific factors that improve the production process performance are manifold and largely unknown. Nonetheless, also plasmid based co-expression of key factors comes with a burden which increases further by the number of expressing gene candidates, while direct modulation of endogenous key factors requires extensive cloning expenditures and time. These efforts become especially noteworthy when considering that the factors to be controlled might vary for any given host strain, process condition, media, and gene of interest and often do not follow a rational design (3,11). Therefore, generic tools that enable multiplexed up-and down-regulation of endogenous genes in a high-throughput mode would enable fast screening and convenient access of critical factors. **C**lustered **R**egularly **I**nterspaced **S**hort **P**alindromic **R**epeats **a**ctivation (CRISPRa) and **i**nterference (CRISPRi) provide the basis to modulate gene regulatory networks without extensive cloning expenditures and are meanwhile well-established tools in eukaryotes for transcriptional up-and down-regulation (13–16). In principle, a deactivated Cas9 (dCas9) mutant that is devoid of its endonuclease activity is either linked to an activator that enhances transcription (CRISPRa) or blocks the binding or elongation of the RNA polymerase (RNAP) respectively (CRISPRi).

**Figure 1.**
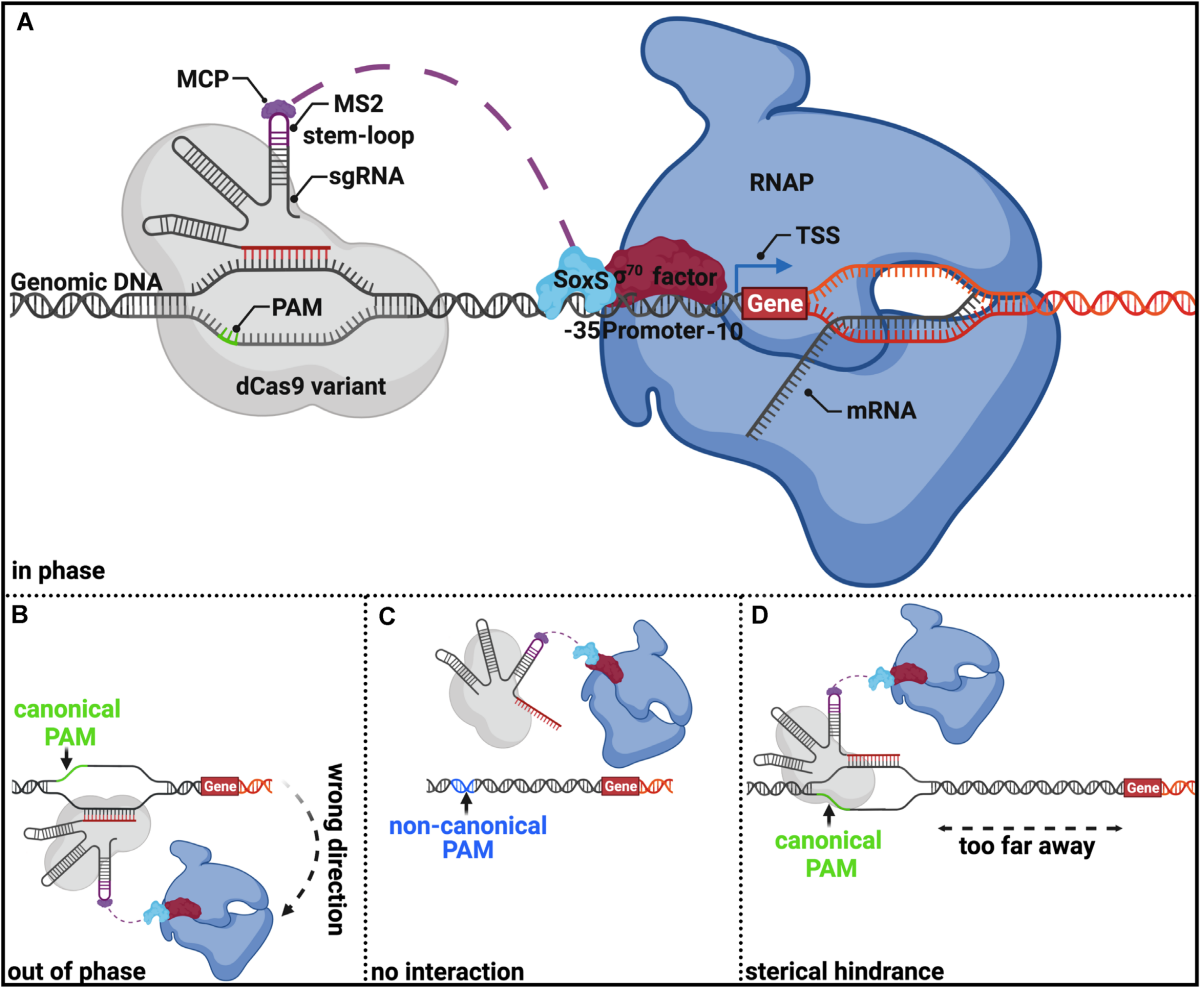
General composition and limitation of a CRISPRa-construct consisting of a dead Cas9 protein variant (dCas9, dxCas9 (3.7), or dSpRY), with an MS2 coat protein (MCP) fused to transcriptional activator SoxS. (**A**) CRISPRa-construct is recruited to a particular upstream promoter region within the up-regulative window of approx. 70–90bp upstream of the TSS. This target site must be adjacent to a canonical PAM sequence (NGG) in the case of conventional Cas9 and allow proper spatial orientation of the transcriptional activator in phase with the target promoter. SoxS interacts with the conserved region 4 of the σ factor 70 family (in this example with σ70 in red) as well as with the α subunit of the core RNAP (blue) and build thereby the RNAP holoenzyme, which initiates transcription of the corresponding target promoter. (**B**) No transcriptional up-regulation will occur when targeting sites within the up-regulative window but with imperfect spatial orientation out of phase relative to the promoter. This is most likely attributed to the helical structure of the DNA, reoccurring every 10–11 bp. The graphic depicts the situation of a 5bp shift from the ideal target site. (**C**) Non-canonical PAM sites adjacent to the target site cannot be recognized by conventional Cas9; thus, no interaction of the CRISPRa construct and the target site will occur. (**D**) Targeting sites outside the up-regulative window sterically hinder the interaction of the recruited RNAP holoenzyme with the promoter.

Bikard *et al.* were the first to use CRISPRa in bacteria by fusing the RNAP-ω subunit to dCas9, originating from *Streptococcus pyogens*, to recruit the RNAP-holoenzyme to trigger the transcriptional up-regulation of a synthetic promoter (17). However, this method requires RNAP-ω deficient strains to be functional, which are significantly hampered in their growth properties (18). More recently, anti-sigma factor phage protein AsiA, phage shock protein PspF, the N-terminal domain of the α-RNAP subunit (α-NTD), and a mutant variant of superoxide stress gene activator SoxS have been successfully used to induce transcriptional activation in bacteria (19–24). However, AsiA interacts solely with σ70-dependent promoters, which mainly control housekeeping genes and narrow its applicability (21,22,25). Being dependent on just one promoter class becomes particularly problematic when considering that key factors for recombinant co-expression, such as chaperones or proteases, are often under the control of alternative sigma factors (26). In addition, overexpression of AsiA impairs cell growth unless its toxicity could be ameliorated by directed evolution (22,25,27). On the other hand, PspF is restricted to the activation of σ54-dependent promoters, which only account for roughly 3% of the entire genome (20,28). CRISPRa-systems using the α-NTD or SoxS as an activator seemed to be the most versatile for up-regulation of endogenous genes since they interact with the σ region 4 (conserved among the σ-factor 70 families) as well as the core RNAP (29–32). These attitudes are important to up-regulate a variety of promoter classes. Up-regulation of alternative σ-factor promoters has already been shown for SoxS (20). The CRISPRa-system using SoxS as an activator includes a scaffold RNA (scRNA), which is a modified version of a gRNA containing an MS2 RNA stem-loop at its 3’ end. This MS2 stem-loop interacts with the corresponding MS2 coat protein (MCP) fused to transcriptional activator SoxS, which recruits the RNAP holoenzyme to a promoter of choice (Figure 1A). To prevent potential off-target effects at endogenous SoxS gene targets, a mutant variant of SoxS (R93A/S101A), decoupling its DNA binding activity from its transcriptional activation function, was developed (19,20). Thus, the SoxS-based CRISPRa construct seems the most appropriate for generic gene activation in bacteria due to the abovementioned features. However, a limitation that probably all bacterial activators share is their sensitivity concerning the distance and spatial orientation toward the corresponding promoter. Bacterial activators possess a very sharp, DNA phase-dependent spacing requirement, demanding an ideal spatial orientation towards their cognate promoter, and are highly sensitive even for slight deviations (19,20,24,33–36). It is hypothesized that this phase dependency is attributed to the geometry and helical structure of the double-stranded DNA, leading to crests and troughs behavior reoccurring every 10–11 bp, which matches exactly a full turn of the helical DNA. Therefore, CRISPRa is only functional at particular sites upstream of the transcriptional start site (TSS) and must be in phase with the promoter (Figure 1A). However, the optimal target positioning apparently varies among different genes (20). When targeting a position just a few nucleotides away from the ideal up-regulation site, the CRISPRa complex will be oriented out of phase. This impedes the interaction of the recruited RNAP holoenzyme with the corresponding promoter (Figure 1B). An additional requirement for binding of the CRISPRa complex is the presence of a protospacer adjacent motif (PAM), consisting of an NGG base triplet. Native Cas9 or dCas9 only unwinds DNA at NGG sites and enables the incorporated guide RNA (gRNA) to build an R-loop formation with its target DNA. This elongates the dwell time of Cas9 in place of its target site (Figure 1A) (37). Targeting sites without adjacent NGG PAMs do not allow DNA unwinding. Thus, no activation occurs (Figure 1C). Studies have shown that in the case of SoxS, the canonical NGG PAM must be located within the up-regulative window of approximately 70 to 90 bp upstream of the TSS (19,20,23,24). Otherwise, steric hindrance prevent interaction with the target promoter (Figure 1D). So far, the PAM stringency has made it impossible to up-regulate most endogenous genes with the current CRISPRa constructs since they require a canonical NGG PAM sequence precisely at sites where the distance and phase allow transcriptional activation. Other researchers tried to bypass these limitations by using a mutated version of dCas9 called dxCas9 3.7 (referred to as dxCas9 from now on), capable of detecting at least some non-canonical PAMs (NGN, GAA, GAT, and CAA have been reported), providing a CRISPRa-system with broader applicability (20,38). However, also these systems do not provide sufficient freedom to bind arbitrary target sites independent of their PAM sequence, which is necessary to be functional. Recently a new PAM independent Cas9 variant called SpRY, originating from *S. pyogenes*, was engineered by Walton *et al.*, detecting NRN and to a lesser extent NYN PAMs (39). During the time course of our experiments, other researchers have successfully implemented a dead variant of SpRY (dSpRY) for PAM flexible transcriptional down-regulation (CRISPRi) in *E. coli* and *Saccharomyces cerevisiae*, as well as for gene activation in rice (39–41). Inspired by the work of the Zalatan lab utilizing SoxS for CRISPRa-based gene up-regulation and the development of the PAM independent variant SpRY by Walton *et al.*, we combined both strategies and engineered a PAM independent CRISPRa tool, which we designated **SMS** (d**S**pRY-**M**CP-**S**oxS). The performance of SMS to up-regulate genes under defined conditions was compared with current SoxS-based CRISPRa-systems, using either native dCas9 or mutant variant dxCas9 (19,20,23,42,43). We could successfully demonstrate that CRISPRa-SMS is a PAM independent gene up-regulation tool in *E. coli* and thus provides unlimited access to accessible DNA target sites required for the ideal spatial orientation of the transcriptional activator towards a promoter. Moreover, we also showed down-regulation and multi-gene expression control with SMS at non-canonical PAMs.

## MATERIALS AND METHODS

### Bacterial strains

The chemical competent bacterial strain *E. coli*-DH5α from New England Biolabs (NEB, USA) was used throughout this study as expression hosts for all reporter gene based experiments, except otherwise mentioned. We used *E. coli*-DH5α for reporter based up-regulation studies since the level of up-regulation of reporters is indirectly estimated by fluorometric analysis of the translated product. By using Lon and OmpT protease deficient *E. coli-DH5*α as an expression host, we wanted to counteract red fluorescent protein (RFP) accumulation and thereby making the test system more robust against misinterpretations. The genome-integrated green fluorescent protein (GFP) producing *E. coli* strain MG1655 was a gift from the Zalatan lab. *E. coli* BL21(DE3) strain, used for RT-qPCR studies for *fkpa*, *poxB* and *surA*, was purchased from NEB.

### Plasmids and plasmid assembly

For plasmid based CRISPRa/i studies, we used a dual plasmid system accordingly with preceding studies by Zalatan *et al.* (19,20,23). The CRISPRa construct was either present on plasmid pCD565 (addgene#153027) or pCK005.6 (addgene#153025) and RFP as the target gene has been present on plasmid pJF076Sa (addgene#113322) (Supplementary Figure S1A). For CRISPRi experiments, we replaced the weak RFP promoter BBa_23117 on plasmid pJF076Sa against the strong promoter BBa_J23119 via Gibson assembly and exchanged the scRNA on the corresponding CRISPR plasmid to a gRNA lacking the MS2 loop via PCR. A list of all scRNAs and gRNAs used in this study can be found in Supplementary Tables S2-S3. The exchange of RFP promoter BBa_J23117 to *extracytoplasmic Spy* (*ECS*) promoter in plasmid pJF076Sa was completed by PCR overhang amplification and subsequent ligation. The *ECS* promoter sequence was derived from ecocyc (https://biocyc.org/ECOLI/NEW-IMAGE?type=OPERON&object=TU-8381). Target plasmid pJF076Sa contains a 240 bp extended upstream promoter region (J1) with multiple canonical PAM sequences, reoccurring every 10 bp on both strands (Supplementary Figure S1A). To permit single base pair resolution with CRISPRa, we generated a library of pJF076Sa with 1 to 10 further base pairs incorporated directly upstream of the promoter, which shifts the PAM sequences spatially further away from the corresponding promoter and thus allows to screen 10 positions with one scRNA (Supplementary Figure S1B). The GFP and RFP encoding plasmid pCD002 (addgene#113313) was used for GFP up-regulation and simultaneous up- and down-regulation studies. To use plasmid pCD002 in *E. coli*-DH5α, we exchanged the R6K-ORI to SC101-ORI via Gibson assembly. Plasmid pJF076Sa has been used as a template for SC101-ORI. A list of RFP/GFP promoter sequences can be found in Supplementary Table S1. All plasmids used in this study originated from the Zalatan lab and were purchased by addgene (Watertown, USA) https://www.addgene.org/Jesse_Zalatan/. The SMS encoding CRISPRa plasmid contains a codon-optimized and catalytic inactive D10A and H840A mutated variant of Cas9 protein SpRY, which was synthesized by IDT as two gblocks and replaced the dxCas9 variant in pCD565 by Gibson assembly. The codon optimized nucleic acid and amino acid sequence of dSpRY can be found in the Supplementary Data. Consecutive SoxS fusion chain were build according to A. Scior *et al.* (44), using XmaI (TypeIIP) and BbsI and BsmBI (TypeIIS) restriction enzymes. The RFP PAM-library was generated by PCR overhang amplification of a degenerated NNN codon at position –81 upstream of the TSS of RFP of plasmid pJF076Sa and subsequent colony PCR screening. Individual colonies were Sanger sequenced at Mycrosynth AG. A list of all plasmids and combinations used throughout our experiments can be found in Supplementary Tables S5–S11.

### Plate-reader measurements

Biologically independent single colonies were isolated and grown overnight for 16 h at 37°C, and 220 rpm in 1 ml lysogeny broth (LB-medium) supplemented with appropriate antibiotics for plasmid pJF076Sa and pCD002 (25 μg/ml ampicillin), and pCD565 (25 μg/ml chloramphenicol). Afterward, 150 μl were transferred into a flat, transparent bottomed, black 96-well microtiter plate from Corning® (#3603) for RFP or GFP detection, as well as optical density (OD) measurements. All measurements were done in Tecan infinite 200Pro plate-reader and analyzed with Tecan i-control software version 1.10.4.0. The excitation wavelength for RFP was 540 nm, and the emission wavelength was 600 nm. The excitation wavelength for GFP was 485 nm, and the emission wavelength was 528 nm. The same 150 μl samples have been used for OD measurements in the Tecan infinite 200Pro plate-reader at 600 nm.

### Reporter gene fold change calculations

All detected reporter gene signals were normalized by the optical density of the corresponding sample and subsequently compared to a normalized off-target control. We determined at least one doubling in reporter signal intensity as a relevant threshold for plasmid-based up-regulation throughout this study due to possible variations in plasmid copy numbers (49–54).

### Fed-Batch-like cultivation in a microbioreactor system

Microscale cultivations were performed in the BioLector system (m2p-labs GmbH, Germany) as described by Fink *et al.* (45,46). with some modifications. Cultivations were performed in fed-batch-like mode in 48-well Flowerplates® (m2p-labs). For this purpose, an overnight culture in LB medium with appropriate antibiotics was seeded. On the next day, an aliquot of 50 μl was inoculated into a 750 μl synthetic Feed in Time (FIT) fed-batch medium containing glucose and dextran as carbon sources (m2p-labs) (45). Immediately before inoculation, 1% (v/v) of the glucose-releasing enzyme mix (EnzMix) was added. The hydrolytic enzyme (glucoamylase) cleaves off glucose residues from solubilized glucose polymer dextran. The enzyme activity controls the cells' growth rate, and the enzymatic glucose release mimics the substrate feed of lab-scale fermentation processes. Bacterial growth was monitored in the microbioreactor via a backward scattered light measurement at 620 nm. Where applicable, RFP was measured with excitation wavelength at 580 nm and emission at 610 nm. RFP values have been normalized against backward scattered light and compared against off-target samples to determine the fold change in RFP up-regulation. The cycle time for all parameters was 15 min. The signals were converted by the BioLector software (BioLection 2.2.0.3). Gas-permeable sealing films (m2plabs) were used to ensure aseptic conditions and reduce evaporation. The humidity in the incubation chamber was controlled (%rH ≥ 85%), and the shaking frequency was 1400 rpm.

### RNA Isolation, reverse transcription and RT-qPCR

Biologically independent single colonies have been isolated and grown overnight at 37°C and 220 rpm in 5 ml lysogeny broth (LB-medium), supplemented with appropriate antibiotics for plasmid pCD565 (25 μg/ml chloramphenicol). On the next day, we made 1:100 dilutions in 5 ml LB-medium and incubated the cells at 37°C at 220 rpm until the OD of 1 was reached (∼4 h). Afterward, we used 1 mL of the culture and added 0.5 ml of 5% buffer-equilibrated phenol [pH 7.4] in ethanol as mRNA stop-solution before centrifugation (2 min, 15 000 × g), flash freezing in LN2, and storage at –80°C. We started the RNA isolation on the same day using the total RNA miniprep kit from Zymo research (#R2014). 2 μg of isolated RNA were applied for reverse transcription using SuperScript™ IV Reverse Transcriptase from Invitrogen. The RT-qPCR analysis have been performed in accordance to the MIQE guidelines http://rdml.org/miqe.html (47). For this purpose, we pooled an aliquot of all reverse transcribed samples and prepared a 1:10 dilution series to optimize the RT-qPCR assay. This dilution series was used to create a standard curve for all testing primers and fulfill the hallmarks of an optimized qPCR assay. The results of the RT-qPCR optimization run can be found in the Source Data File. RT-qPCR assays were prepared in 20 μl reactions according to iQ™ SYBR® Green Supermix guidelines with 0.1 ng to 1ng of sample cDNA. A list of RT-qPCR primers can be found in Supplementary Table S4. All measurements have been done in the MiniOpticon™ system from Biorad and analyzed with the CFX Manager™ software version 3.1.1517.0823. Fold changes for RT-qPCR were determined by the ΔΔCT method (48).

### Statistical analysis

Statistical significance was calculated using one- or two-tailed ANOVA performed via Graphpad Prism 9 for macOS, Version 9.3.1 (350), December 7, 2021. All experiments were performed with at least *n*  =  3 biologically independent samples. Data and results about all statistical analyses can be found in the Supplementary File Statistics.

## RESULTS

### SMS is functional at canonical PAMs but yields in lower up-regulation

As a proof of concept, we wanted to validate the functionality of the current CRISPRa-system in *E. coli*-DH5α, using the R93A/S101A mutant variant of SoxS as a transcriptional activator. This d**x**Cas9-**M**CP-**S**oxS based CRISPRa construct is referred to as **XMS** throughout this study.

**Figure 2.**
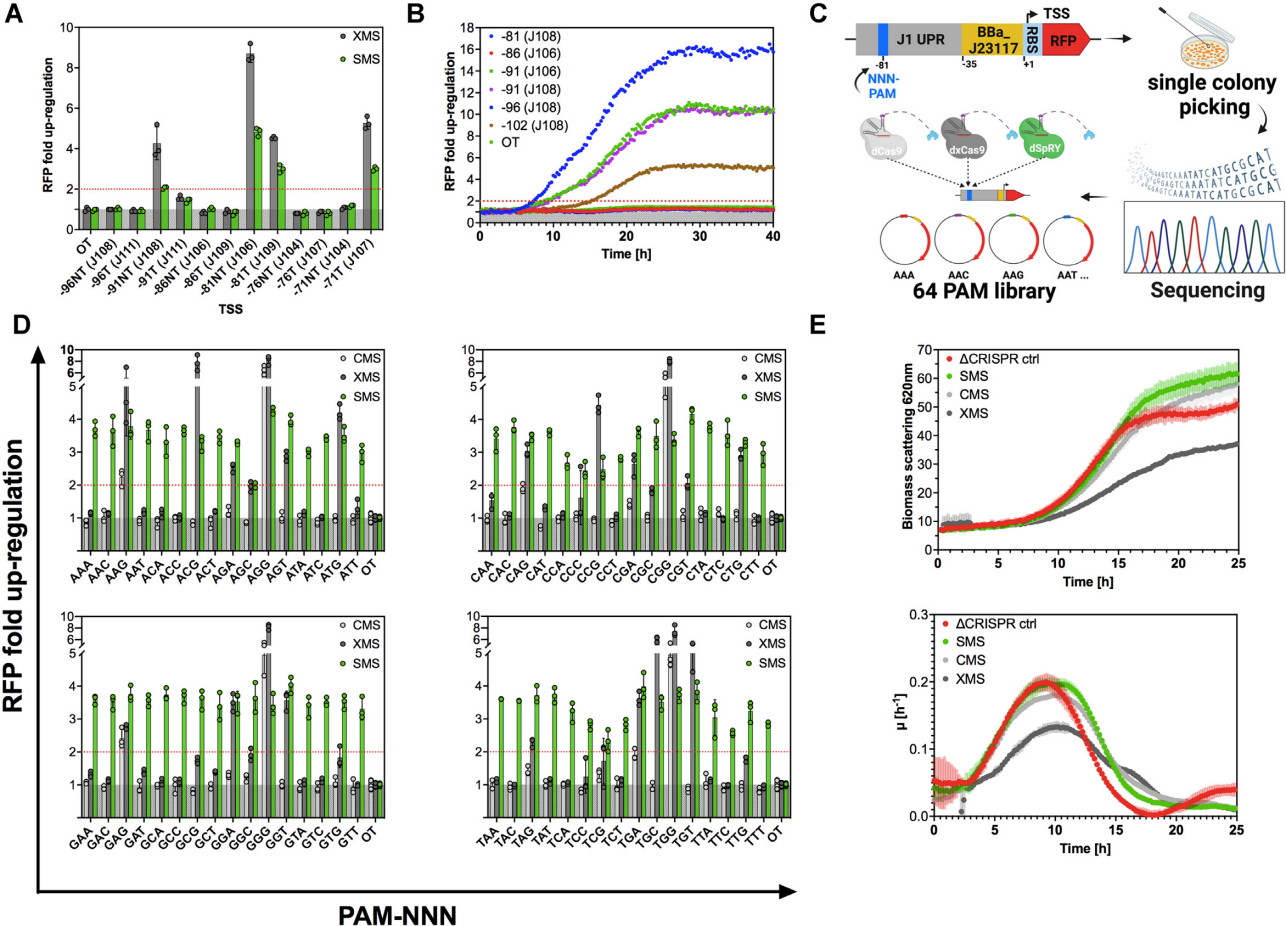
CRISPRa-SMS is a PAM independent up-regulation tool with lower performance at canonical PAM sequences. (**A**) SMS (green) exhibit the same phase-dependent up-regulation pattern as XMS (dark grey), although the overall fold change in up-regulation was lower, which comes most likely with the cost of PAM independency. Data represent mean values, and error bars represent the SD of *n = 3* biological replicates. Two-Way-ANOVA has been performed for all samples. (**B**) Continuous measurements of XMS mediated RFP up-regulation, revealed that the level of up-regulation changed over time, despite sample normalization against the optical density. (**C**) Degenerated NNN-PAM was cloned into the J1 region at position -81 upstream the TSS. Subsequent colony screening was performed to isolate a RFP PAM-library comprising all 64 possible PAMs at position -81. (**D**) MS2/MCP-SoxS based CRISPRa constructs with dCas9 (CMS-light grey), dxCas9 (XMS-dark grey) and dSpRY (SMS-green), have been tested to up-regulate the RFP PAM-library from position -81 upstream the TSS at the NT strand. Considerable up-regulation with SMS at all 64 PAMs could be observed. Up-regulation at 24 PAMs (20 non-canonical) were recorded by XMS and at 8 PAMs (4 non-canonical) by CMS. Data represent mean values and error bars represent the SD of *n*= 3 biological replicates. Two-way ANOVA has been performed for all samples and could confirm statistical significance for up-regulation with SMS independent of the PAM sequence, while for CMS and XMS not. (**E**) Growth kinetics of CMS; XMS and SMS were determined by light scattering analysis at 620 nm in a microbioreactor system. CMS (light grey) and SMS (green) depict similar cell densities compared to no CRISPR control (ΔCRISPR ctrl-red). XMS (dark grey) depicts with a maximal growth rate (μ) of 0.15 h^−1^ slight growth impairments compared to XMS and SMS (μ_max_= 0.2 h^−1^). Data represent mean values, and error bars represent the range of *n*= 3 biological replicates. Results of all statistical analyses are provided in the Supplementary File Statistics. OT = off-target

Plasmid-based red fluorescent protein (RFP) was chosen as the target gene under the control of the weak constitutive promoter BBa_J23117 (iGEM Anderson promoter collection, http://parts.igem.org/Promoters/Catalog/Anderson), which depicts a high dynamic range. According to preceding studies, positions –71, –81 and –91 upstream of the TSS are supposed to be in phase in this experimental setup, while positions –76, –86 and –96 are supposed to be out of phase (compare Figure 1A, B) (19,20,23,55). We targeted both, the template (T) and non-template (NT) strand of these positions and monitored the RFP expression by plate reader experiments 16h after inoculation (Figure 2A and Supplementary Figure S1). Due to possible fluctuations of the plasmid copy number, we decided to define a doubling in reporter gene signal as a threshold throughout this study (49–54). We observed phase-dependent RFP fold changes by XMS that matched well with the data from previous studies (17,19–24).

As we hypothesized that CRISPRa-mediated fold changes of reporter genes might be time dependent due to protein expression, degradation, and accumulation dynamics, we performed cultivations of XMS under fed-batch-like conditions in microbioreactor systems, allowing us to monitor the RFP fold change continuously (56). We could show that the extent of the up-regulated RFP signal was indeed dependent on the measurement timepoint despite sample normalization using the cell density (Figure 2B).

In the next step, we wanted to eliminate the PAM dependency and replaced the dxCas9 variant with the PAM independent Cas9 variant dSpRY and established the CRISPRa construct SMS. For this purpose, we codon-optimized the sequence of Cas9 variant SpRY for expression in *E. coli* and mutated the two catalytically active domains RuvC and HNH at positions D10A and H840A, as ordinarily done to deactivate Cas9 endonucleases (57,58). Afterward, we verified the general functionality of the resulting SMS construct with the same experimental setup as for XMS described above (Supplementary Figure S1). Like XMS, also SMS displayed a DNA phase-dependent up-regulation pattern for RFP, with the highest fluorescence signal at position -81 upstream of the TSS (Figure 2A). As expected, the extent of up-regulation was somewhat lower with SMS since a decreased overall DNA binding capacity of SpRY was previously reported in favor of PAM independency (39).

### SMS is a PAM independent up-regulation tool

To confirm the PAM independency of SMS, we established an RFP PAM library, comprising all 64 possible PAM sequences (4 canonical and 60 non-canonical) at position -81 (Figure 2C). We then examined the up-regulation propensity for the RFP PAM library with MCP-SoxS based CRISPRa systems using either dCas9 (CMS), dxCas9 (XMS), or dSpRY (SMS) (Figure 2D). The level of up-regulation was determined 16 h after inoculation, congruent with the experimental setup in the section above. We observed a 2- to 4-fold up-regulation for SMS regardless of the PAM sequence, while XMS showed noticeable up-regulation of around 20 non-canonical PAMs. CMS resulted in slight up-regulation at non-canonical NAG and TGA PAMs, which fits to previous studies with *S. pyogenes* Cas9 (60–63). However, the up-regulation levels of SMS were somewhat lower than for XMS and CMS at canonical PAMs. To further verify the PAM independent up-regulation propensity of SMS, we decided to continuously monitor four rather low performing PAM sequences (AGC, CCA, CCC, CCG) from Figure 2D, by using the microbioreactor system (Supplementary Figure S3). Congruent to plate-reader measurements but at more extensive fold change levels, we could see up-regulation by SMS at all 4 PAMs (at peaks: AGC 6-fold, CCA 10-fold, CCC and CCG 12-fold). XMS showed stronger up-regulation as SMS at CCG PAM (17-fold), similar up-regulation for AGC (7-fold), but no up-regulation at CCA and CCC PAM. CMS monitoring against AGC, CCA, CCG and CCC did not result in considerable up-regulation above the threshold. Statistical analysis confirmed that SMS depending up-regulation was significant throughout all PAM sequences, while XMS and CMS were not. Results are provided in the Supplementary File Statistics.

### Fusion chain of SoxS does not lead to synergistic enhancement of CRISPRa

Several approaches were conducted in the past to further increase the CRISPRa efficiency as well as to broaden its up-regulative window. The efforts for enhancing CRISPRa efficiency and flexibility (elimination of phase dependency) in bacteria reached out from different scRNA designs including multiple MS2 loops, usage of several scRNAs at once, and longer MCP-SoxS linkers (19,20,22). All of these approaches resulted in little or no success. In mammalian cells, combinations of different, or multiple consecutive transcriptional activators have been successfully used for synergistic CRISPRa enhancements (15,16,59). Therefore, we tried to increase the overall CRISPRa efficiency and flexibility by building a chain of SoxS C- to N-terminal domain fusions (Supplementary Figure S2A). Thereby the number of activators was multiplied for potential synergistic enhancements, which was also thought to be a phase independent design that resembles the SunTag approach in eukaryotes (15). However, we saw a drop in CRISPRa efficiency rather than an increase using a chain of two and three consecutive fused SoxS activators with XMS (Supplementary Figure S2B und C). We hypnotized that the N-terminal domain of SoxS might be crucial for its functioning and thus designed a single N- to N-terminally fused MCP-SoxS activator construct. The data revealed indeed a loss of activator functioning when SoxS is N-terminally fused (Supplementary Figure S2B). Instead of further increasing the level of CRISPRa efficiency and flexibility, we decided to focus our research on in deep investigations on PAM intendency of SMS.

### Authentic SMS expression does not impair cell growth

Overexpression of CRISPR systems has been shown to induce a cell burden leading to a variety of altered cell stages, such as growth defects or morphological changes (64–66). In order to circumvent toxic side effects, we preferred to use the authentic native *S. pyogenes* promoter, rather than a constitutively and/or strong synthetic promoter. To examine if SMS expression leads to impaired cell growth in our setup, we monitored cell growth by light scattering analysis in a microbioreactor system of three different CRISPRa constructs using either (i) dCas9 (CMS), (ii) dxCas9 (XMS) and (iii) dSpRY (SMS), together with the MCP-SoxS fusion protein as activator and an off-target scRNA for its assembly. A noCRISPR control (ΔCRISPR ctrl) was used as reference. The recorded growth curves indicate that the CRISPRa systems we expressed, do not show impaired cell growth upon CMS or SMS expression (Figure 2E). However, a reduction in growth kinetics was observed for XMS expression.

### SMS enables transgene and endogenous up-regulation at non-canonical PAMs

**Figure 3.**
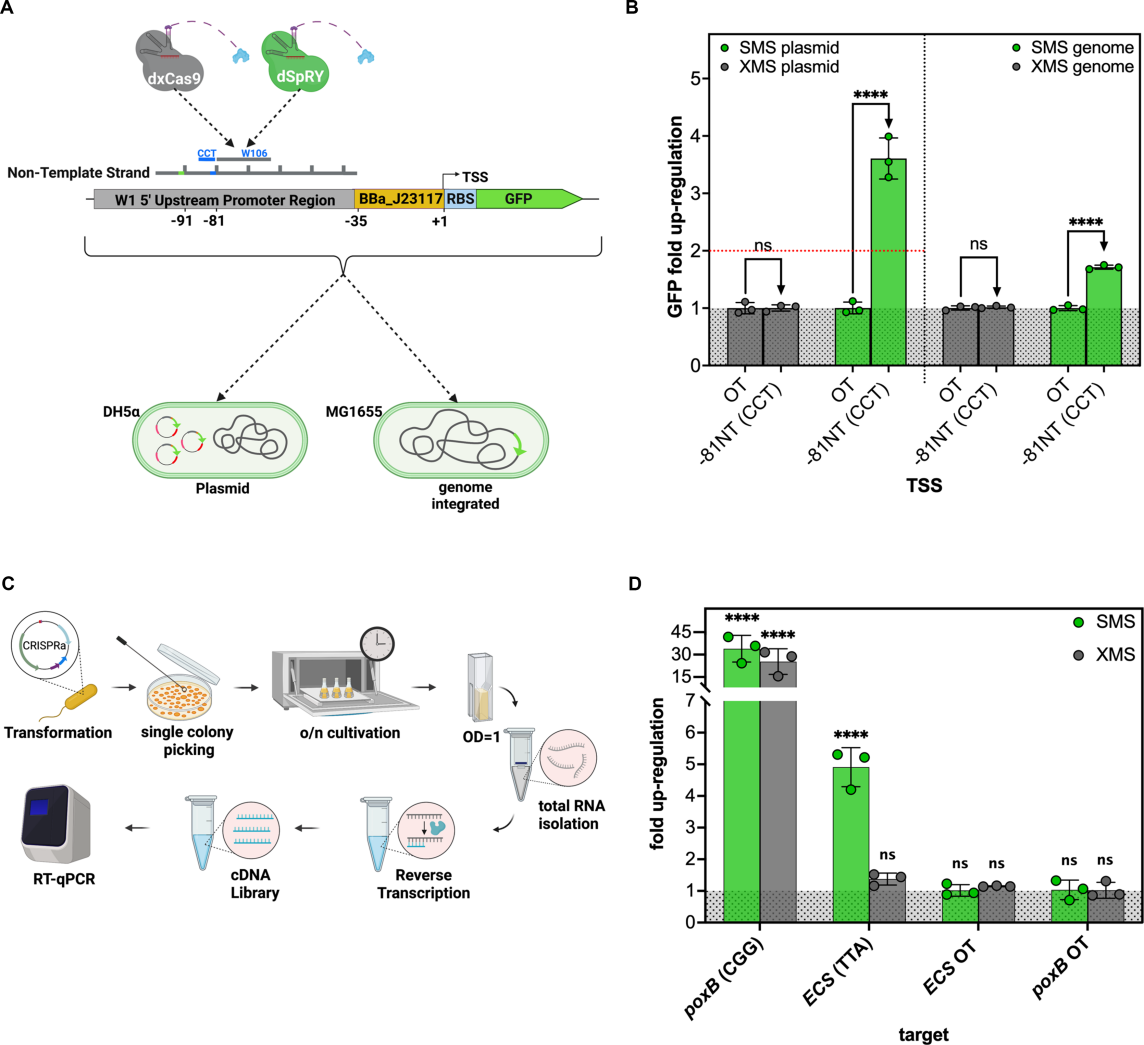
SMS is functional at non-canonical PAMs during transgene and endogenous gene up-regulation. (**A**) To verify transgene up-regulation with SMS at non-canonical PAMs, we used *E. coli* MG1655 with genome-integrated GFP which is under the control of promoter BBa_J23117. For the up-regulation of transgenic GFP, we used XMS and SMS and targeted position -81 bp which is adjacent to non-canonical CCT. For comparison, we also targeted the original GFP plasmid on the same position in *E. coli*-DH5α. (**B**) SMS mediated up-regulation on plasmid (3.61-fold) and genome-integrated GFP (1.71-fold) resulted in considerable higher GFP levels compared to its off-target controls (OT), while XMS does not. All measurements were performed in *n*= 3 biological replicates (**C**) Afterwards, we performed RT-qPCR to verify endogenous up-regulation propensities with XMS and SMS at non-canonical PAMs. We chose endogenous *ECS* as a model protein and targeted it at position -80NT which is adjacent to a TTA PAM. For this purpose, we transformed *E. coli*-DH5α with CRISPRa plasmids and inoculated them into LB-medium with appropriate antibiotics, followed by incubation overnight. On the next day, we diluted the suspension 1:100 and let them grow until an OD of 1. Total RNA was then isolated and reverse transcribed followed by RT-qPCR measurement. (**D**) RT-qPCR studies at position -80 NT revealed statistically significant up-regulation of *ECS* with SMS (4.91-fold) and no up-regulation with XMS (1.37-fold). The positive control *poxB* could be up-regulated by both, SMS and XMS, which verifies their general functionality. All RT-qPCR datapoints were performed in *n*= 3 biological replicates, measured in technical replicates according to MIQE guidelines. Two-way ANOVA has been performed for plate-reader and one-way ANOVA for RT-qPCR experiments, confirming the statistical significance up-regulation of SMS. Results of all statistical analyses are provided in the Supplementary File Statistics. ****P< 0.0001. OT = off-target control

To prove the functionality of SMS for transgenes at non-canonical PAMs, we tested the magnitude of up-regulation of a genome integrated weakly expressed green fluorescent protein (GFP) (Figure 3A). For comparison, we also tested the extend of GFP up-regulation on the original plasmid (Figure 3A), which contains GFP under a weak constitutive promoter and RFP under a strong constitutive promoter. Experiments for genome integrated GFP up-regulation have been conducted in *E. coli* MG1655 and plasmid based GFP up-regulation in *E. coli*-DH5α. Both of which are K12 derivates. We targeted position -81NT upstream of the TSS of GFP using scRNA W106, which was adjacent to a non-canonical CCT PAM. As expected, we observed considerable up-regulation of GFP (3.61-fold) above the doubling threshold when targeting the plasmid and a somewhat lower level for the genome integrated cassette (1.71-fold) (Figure 3B). The differences in fold changes between the plasmid and the genome integrated up-regulation approaches might be attributed to the target gene copy number. Nonetheless, the results indicated PAM independent functionality of SMS on plasmid systems and at endogenous genes.

As a next step, we wanted to up-regulate an authentic endogenous gene at a non-canonical PAM-site in *E. coli*-DH5α. We chose *extracytoplasmic chaperon Spy*, referred to as *ECS* (not to be confused with Cas9 variant SpRY), and the pyruvate dehydrogenase gene (*poxB*) as model proteins. *ECS* is a periplasmic chaperon, which acts as a holdase to prevent protein aggregation and as a foldase to assist protein folding simultaneously (67–69). *poxB* was shown to be responsive to XMS dependent up-regulation at position -91NT upstream of the TSS in a previous study, using a promoter library collection with endogenous gene cassettes (20). Therefore, we targeted authentic endogenous *poxB* at position -91NT and used it as a positive control to verify the general functionality of XMS and SMS. To determine the ideal up-regulation site for *ECS*, we first used the RFP plasmid pJF076Sa from in Figure 2A, B and D and replaced its RFP promoter (BBa_J23117) against the endogenous promoter sequence of *ECS* (Supplementary Figure S4A). We then generated a library with 1 to 10bp incorporated upstream the *ECS* promoter and screened position –73 to –82 with SMS and XMS, targeting canonical PAMs on the NT strand. (Supplementary Figure S4A). SMS and XMS displayed a similar up-regulation pattern, peaking at position –80 with an ∼5-fold overexpression for the *ECS* promoter-driven RFP (Supplementary Figure S4B). However, endogenous *ECS* accommodates a non-canonical TTA sequence at position –80 upstream of the TSS, which can thus be only targeted by SMS. We then performed reverse transcription quantitative PCR (RT-qPCR) studies according to MIQE guidelines, to verify the functionality of SMS for up-regulating the endogenous *ECS* at non-canonical TTA PAM (Figure 3D) (47). The transcriptional fold change of *poxB* and *ECS* after XMS and SMS up-regulation was determined by comparison against an off-target control. As expected, we observed strong transcriptional up-regulation of *poxB* for both constructs (SMS 33.94-fold, XMS 25.29-fold), due to the presence of a canonical CGG PAM, at an optimal position relative to the promoter (Figure 3D). *ECS* was shown to be roughly 5-fold up-regulated by SMS (4.91), while XMS dependent up-regulation displayed a statistically no relevant foldchange (1.37) (Figure 3D). To demonstrate endogenous up-regulation also in an *E. coli* production strain, we targeted two further chaperons, namely *fkpa* and *surA* in *E. coli*-BL21(DE3). Although we were not seeking the optimal positioning for *surA* and *fkpa* up-regulation, we expected it to be around position -80NT upstream of the TSS. Therefore, we targeted both at position –80NT, which is adjacent to a non-canonical PAM site. A 2.4-fold up-regulation of *surA* and a 3.4-fold up-regulation of *fkpa* was achieved (Supplementary Figure S5). Again, we used *poxB* as a positive control at the -91NT position, leading to an 11-fold up-regulation (Supplementary Figure S5). Thus, we demonstrated that SMS can be used for transgene and endogenous gene up-regulation at non-canonical PAM sites. RT-qPCR primer efficiencies and melt curve analysis can be found in the Source Data File.

### SMS enables down-regulation at non-canonical PAMs

**Figure 4.**
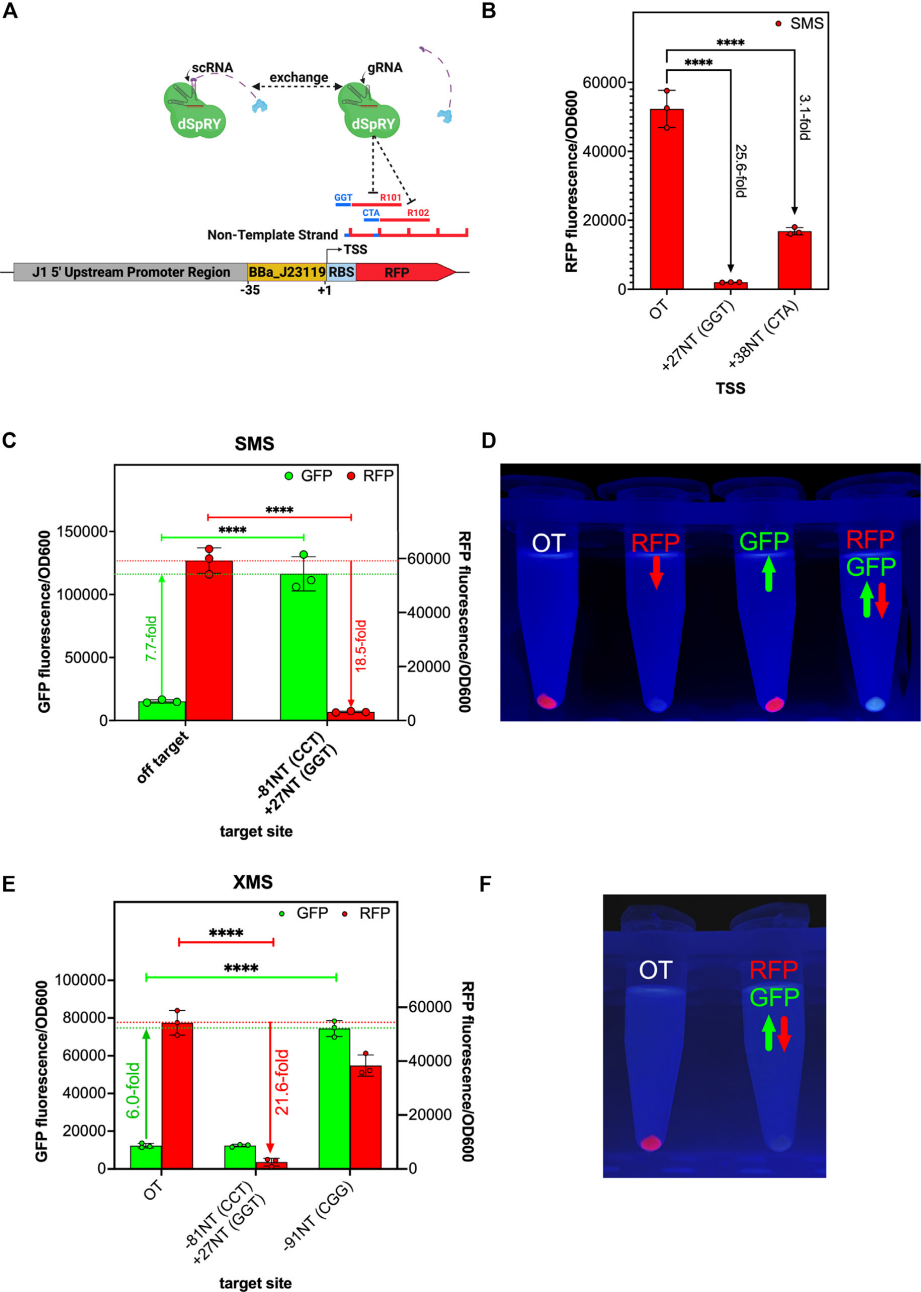
SMS is functional for down-regulation and multi-gene expression control at non-canonical PAMs. (**A**) We exchanged the scRNA to a gRNA, lacking the MS2 loop and thus, do not interact with the MCP-SoxS activator. To measure the magnitude of down-regulation, gRNAs R101 and R102 were targeting the RFP coding sequence at position +27 (GGT PAM) and +38 (CTA PAM). (**B**) RFP signal was down-regulated several orders of magnitude at non-canonical PAM sites. gRNA R101 resulted in more efficient down-regulation than R102, which might be attributed to better gRNA binding affinity at R101 site. (**C**) Concurrent expression of scRNA W106 and gRNA R101 with SMS lead to simultaneous up-regulation of weakly expressed GFP (7.7-fold) and down-regulation of strong expressed RFP (18.5-fold) (**D**) Strong up- and down-regulative effects lead to phenotypic changes in cell appearance, when using SMS. Left: Off-target control (strong expressed RFP and weak expressed GFP), middle-left: down-regulated RFP and low expressed GFP (Figure 4B), middle-right: up-regulated GFP and strong expressed RFP (Figure 3B), right: down-regulated RFP and up-regulated GFP. (**E**) Simultaneous up- and down-regulation is not functional using XMS. Successful down-regulation with XMS using R101 could be achieved, since it depicts activity at GGT PAM. However, no up-regulation can be noticed when targeting GFP using W106 on a CCT PAM. To ensure proper XMS functionality, we up-regulated GFP using W108 at canonical CGG PAM. (**F**) Left: off-target control, right: simultaneous GFP up- and RFP down-regulation (no change from red to green cell pellet visible). Data represent mean values and error bars represent the SD of *n* = 3 biological replicates. Two-way ANOVA has been performed confirming the statistical significance of RFP down-regulation and GFP up-regulation. Results of all statistical analyses are provided in the Supplementary File Statistics. ****P< 0,0001. OT = off-target control

In most situations, down-regulation of genes is not genuinely restricted by PAM dependency. However, for some specific gene regulatory purposes, as for sophisticated genetic ciruits, PAM independent interference can be relevant. Therefore, we wanted to confirm the down-regulative capacities of SMS at non-canonical PAMs and targeted a strong expressed plasmid-based RFP at its coding sequence on position +27NT, and in a separate experiment a few bases further downstream at +38NT (Figure 4A). Both target sites are adjacent to non-canonical GGT and CTA PAMs, respectively. For the purpose of down-regulation, we exchanged the scRNA to a gRNA, lacking the artificial MS2 loop, which abolishes the interaction of the MCP fused activator SoxS (Figure 4A). We could demonstrate extensive down-regulation of RFP at both target sites (Figure 4B). However, down-regulation at position +27NT was much more effective, leading to a complete loss of fluorescence signal (Figure 4D). Based on these observations, we conclude that down-regulation is also PAM independent for SMS.

### SMS enables multi-gene expression regulation at non-canonical PAMs

One of the greatest advantages of CRISPRa and CRISPRi is their potential for multi-gene expression regulation. Due to the somewhat lower overall up-regulation potential of SMS in favor of its PAM independency, it was questionable if multi-gene expression studies could be performed with SMS at non-canonical PAMs. Therefore, we designed a dual scRNA/gRNA SMS construct for simultaneous up-regulation of a weak expressed GFP and down-regulation of a strong expressed RFP, both encoded on one plasmid (Supplementary Figure S6A). For GFP up-regulation, we targeted position -81NT (CCT PAM) and position + 27 (GGT PAM) for RFP down-regulation (Supplementary Figure S6A). We thereby observed a strong 7.7-fold up-regulation of GFP and an 18.5-fold down-regulation of RFP (Figure 4C). This simultaneous up-and-down-regulation at non-canonical PAMs led to a phenotypic color switch from a red to a green cell pellet compared to its off-target control sample (Figure 4D). We have shown in Figure 2D that XMS showed activity at non-canonical GGT PAM, but no activity at CCT PAM. As expected, we could not see a phenotypic switch from red to green cells when repeating the experiment with XMS, but instead observed solely a strong down-regulation of RFP, leading to a colorless, non-fluorescent cell pellet (Figure 4E and F). To ensure the proper function of XMS in this experiment, we targeted GFP at position -91NT, adjacent to canonical CGG PAM and observed a 6-fold up-regulation (Figure 4E). This result strikingly demonstrates the abilities and benefits of SMS for multi-gene expression control at non-canonical PAMs.

## DISCUSSION

CRISPRa-based transcriptional up-regulation in bacteria requires stimulation from a target site located at a proper distance and in phase with the target promoter to be functional. Even slight deviations from it can fully abolish its up-regulative propensity (Figure 2A and Supplementary Figure S4B) (19,20,23,36). Thus, for endogenous CRISPRa applications, the presence of a NGG PAM, adjacent to a functional CRISPRa target site, is a prerequisite but rarely the case. The CRISPRa constructs reported so far are all PAM dependent to a certain extent, which hinders the binding to functional CRISPRa target sites and prohibits up-regulation for most endogenous genes. Our construct, composed of dSpRY-MCP-SoxS referred to as SMS, has been used to up-regulate a RFP library consisting of all 64 possible PAM sequences and demonstrated thereby its PAM independency. Although SMS depicts a lower fold change in up-regulation at canonical PAMs compared to previously reported CMS and XMS constructs, it maintains its up-regulative potential at non-canonical PAM sequences (Figure 3B). The lower fold change of SMS at canonical PAM sites might be explained by self-targeting events, thus blocking its coding sequence, which comes hand in hand with its PAM independency. Self-targeting of PAM independent CRISPR constructs seems to be unavoidable and cannot be circumvented using authentic DNA encoding scRNAs or gRNAs (70,71). We tried out to mitigate this event by building a consecutive SoxS chain for potential synergistic effects without success. It seemed that the N-terminal domain of SoxS is indispensable for its activator functioning (Supplementary Figure S2). However, another authentic or synthetic activator might exist which possesses a higher affinity to the RNAP and/or might allow the design of more sophisticated arrangements which facilitates synergism. Worth noticing is that this apparently lower efficiency of SMS at canonical PAM sites did not lead to lower up-regulation level for *poxB* in RT-qPCR studies (Figure 3D), nor for plasmid-based RFP under the control of an endogenous *ECS* promoter (Supplementary Figure S4B). We assumed that the amount of SMS-mediated RNAP recruitment was sufficient (saturated) for these promoters to exploit their whole dynamic range. Other promoters, like BBa_23117 might need higher amounts of RNAP to exhaust their limits. If possible, we encourage to use CMS or XMS at sites where these constructs performed better compared to SMS in Figure 2D. This would, however, limit the possibilities for wide-ranging CRISPRa studies. Another method to enhance the overall CRISPRa efficiency could be the use of multiple scRNA in the close proximity of the ideal target site. Synergistic effects have already been shown for CRISPRa in bacteria (22). SMS would be the perfect candidate for this endeavor, as it can target multiple positions without considering PAM sequences.

Continuous monitoring of CRISPRa treated cultures demonstrated that the observed up-regulation level of fluorescent reporters is time dependent due to accumulative effects, even after sample normalization (Figure 2B and Supplementary Figure S3). Thus, comparative CRISPRa studies with reporter genes are only allowed when all samples are treated by the same conditions and equivalent incubation time. This time dependency is not surprising, considering that fluorescent reporters can have a half-life of above 20 h and will thus be passed on to daughter cells even after several events of cell divisions (72). However, the level of mRNAs will be revoked after cell division due to its shorter half-life of maximal 10–20 min (73). Thus, indirect up-regulation measurements via reporter genes are rather supposed for qualitative analysis of general CRISPRa functionality under certain conditions and only contingently qualified to predict absolute up-regulation fold changes. Growth kinetics of CMS, XMS and SMS showed that the expression of dxCas9 has a slight negative impact on cell growth using the native *S. pyogenes* Cas9 promoter. We detected a decrease in μ_max_ from 0.2 h^−1^ to 0.15 h^−1^ compared to a noCRISPR control (Figure 2E). However, CMS and SMS depicted congruent growth kinetics comparable to noCRISPR control (Figure 2E). Since the dSpRY variant we used in our experiments is codon optimized (see Supplementary Data for nucleic acid sequence), it might be possible that the impairments in cell growth of dxCas9 can be eliminated by codon optimization.

Successful transgenic and endogenous up-regulation at non-canonical and canonical PAM sites (Figure 3B and D, Supplementary Figure S5), suggests an actual generic, PAM sequence independent application of SMS for up-regulating endogenous genes in *E. coli*. We did not test SMS efficiency in other bacterial cell hosts. However, we assume that SMS might also be functional in different prokaryotic species since SoxS is a mediator of the global stress response and belongs to the AraC/XylS family of transcriptional regulators found among the family of *Enterobacteriaceae* (74–77). Its interaction points with the σ-factor region 4 and the core RNAPα-subunit (both well conserved in bacteria) might qualify SoxS even as transcriptional activator for a wider variety of prokaryotic family members (29–32). Indeed, a dCas9-MCP-SoxS (CMS) construct has been successfully applied in *Pseudomonas Putida* (23). In addition, also different Cas9 variants seemed to be functional for CRISPRa purposes in various bacterial species. dCas9 or dxCas9 variants, fused to transcriptional activator AsiA, PspF, α-NTD of the RNAP, or ω-domain of RNAP have been successfully used in *Pseudomonas syringae*, *Klebsiella pneumoniae*, *Salmonella enterica, Streptomyces venezuelae, Bacillus subtilis* (22,36,66,78). These findings, furthermore, underline also the translatability of Cas9 variants among prokaryotes.

Throughout our studies, we could see different extents of up-regulation ranging from roughly 2-fold for genome integrated *GFP* (Figure 3B) to more than 30-fold for endogenous *poxB* (Figure 3D). Albeit, one should consider that the level of transcriptional up-regulation was determined at different layers (reporter genes indirectly via fluorescence; *poxB, ECS, surA* and *fkpa* directly via RT-qPCR). The efficiency of CRISPR mediated up-or down-regulation depends on several parameters, (i) the accessibility and binding efficiency of the gRNA/scRNA at its target site, (ii) the sequence composition between the target site and the corresponding promoter, (iii) the presence or absence of repressors, and (iv) the dynamic range of the promoter (19,20,28). This might explain why some promoters can be easily up-regulated to several orders of magnitude while others respond only moderately.

We also validated the down-regulation capabilities of SMS at non-canonical PAMs and demonstrated a substantial drop in gene expression when targeting the coding sequence of RFP, which was even visible by the unaided eye. We observed a considerably greater extent of down-regulation at position +27 compared to +38 (Figure 4B). Again, the efficiency of CRISPRa or CRISPRi depends on various factors described above. Thus, the gRNA binding efficiency at position +38 might be somewhat weaker compared to position +27. However, both approaches lead to considerable and statistically significant down-regulation of its target gene and demonstrated functionality at non-canonical PAMs.

Multi-gene expression studies of SMS strikingly demonstrated its capacity by simultaneous up-and down-regulation of GFP and RFP at non-canonical PAMs. Surprisingly, the level of GFP was thereby twice as high up-regulated (7.7-fold instead of 3.61-fold) and RFP somewhat lower down-regulated (18.5-fold instead of 25.6-fold) compared to their single-gene expression controls (Figures 3B and 4B). This observation might eventually be attributed to a lower stress level of the host cell since the strong promoter BBa_J23119 controlling RFP was down-regulated and potentially provided more resources to up-regulate the weakly expressed GFP.

Although SMS has been shown to overcome PAM stringency, which provides freedom to operate at any accessible DNA position, the apparent functional CRISPRa target site of an individual gene must be assayed empirically and cannot be determined by simple distance metrics (20). The reason for the differences in functional CRISPRa target sites is unclear yet but might be attributed to slight deviations of the DNA phase at certain distances relative to the promoter. Determining the optimal positioning for each gene of interest is doable but can get readily laborious when multiple gene candidates are to be up-regulated. Thus, a CRISPRa design that is phase independent and therefore tolerant to slight deviations from the ideal functional CRISPRa site is needed for high throughput applications. Surprisingly, it was shown that prolonged and hence more flexible activator fusions did not tolerate deviations from ideal functional target sites (20). A wide-ranging CRISPRa study in bacteria at various genes might help generate a predictive DNA sequence-specific formula. Another approach to tolerate such deviations could be an entirely different interaction design of the activator and Cas9. Kcam *et al.* performed circular permutation studies of Cas9 fused with α-NTD and demonstrated that the optimal up-regulation site depends on the surface patch at which the activator is linked (24). Taken together, SMS provides a generic CRISPRa-system in bacteria, which can bind to any accessible target site without PAM limitations. Thereby, it enables a maximum level of freedom for CRISPRa navigation at endogenous genes and allows design-build-test-learn studies, forward and reverse genetics, or elucidation of the interplay of complex signaling pathways, which are essential for many academic and biotechnological purposes.

## DATA AVAILABILITY

Data points from all figures are provided in the Source Data File. Statistical analysis for all figures can be found in the Supplementary Statistics File. Combinations of plasmids for all experiments can be found in the Supplementary Materials.

All plasmids used in this study originate from the Zalatan lab and can be purchased by addgene (https://www.addgene.org/Jesse_Zalatan/).

## Supplementary Material

gkac804_Supplemental_Files

## Notes

Present address: Christian Doppler Laboratory for Production of Next-Level Biopharmaceuticals in *E. coli*, Department of Biotechnology, Institute of Bioprocess Science and Engineering, University of Natural Resources and Life Sciences, Vienna, 1190 Vienna, Austria.
