# Supplementary material for: CRISPRactivation-SMS, a message for PAM sequence independent gene up-regulation in Escherichia coli
Source: Nucleic Acids Res. 2022 Sep 22;50(18):10772–84. doi: 10.1093/nar/gkac804 (PMC9561276; doi:10.1093/nar/gkac804)
Supplement: gkac804_Supplemental_Files [file gkac804_supplemental_files.zip › Supplementary_Data_NAR-02040-J-2022 2_new.pdf]

CRISPRactivation-SMS, a message for PAM sequence independent gene up-regulation in *Escherichia coli*

Marco Klanschnig[1], Monika Cserjan-Puschmann[1]*, Gerald Striedner[1], Reingard Grabherr[1]

[1] Christian Doppler Laboratory for Production of Next-Level Biopharmaceuticals in *E. coli*, Department of Biotechnology, University of Natural Resources and Life Sciences, Vienna, 1190 Vienna, Austria;

SUPPLEMENTARY DATA

Schematic overview of the design and working procedure for CRISPRa plate-reader experiments

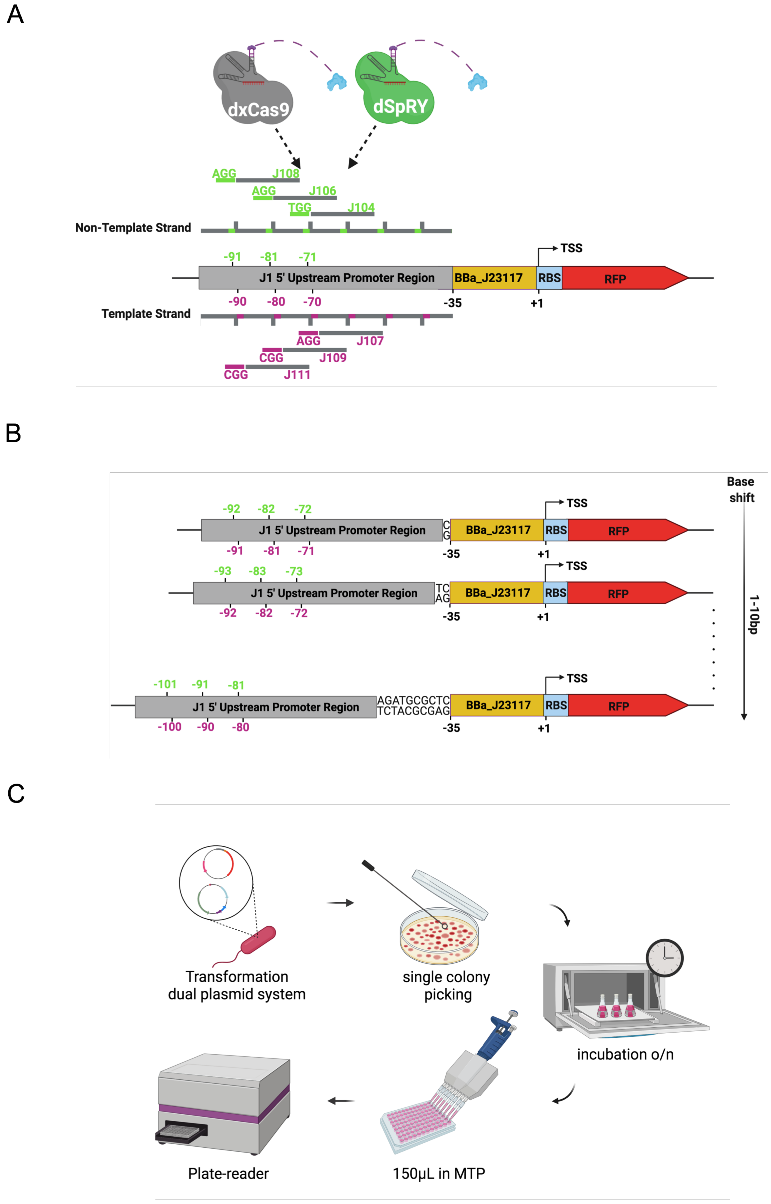

Figure S1 Schematic overview of the design and working procedure for CRISPRa plate-reader experiments: (**A**) To verify CRISPRa based up-regulation, a dual plasmid system was used, consisting of the CRISPRa construct with either dxCas9 (XMS-dark grey) or SpRY (SMS-green) and the RFP target gene. scRNAs J104, J106, J107, J108, J109, J111 and an off-target control were used to target the template (T) and non-template (NT) strand respectively. (**B**) Incorporation of 1 to 10 bp upstream the promoter sequence of RFP, shifts the canonical PAM sequences in the J1 region upstream the promoter and generates thereby a library which allows to screen 10 positions, with only one scRNA. (**C**) After transformation and selection on agar plates with appropriate antibiotics, we picked biologically independent cell colonies for overnight incubation (16h) in liquid media and measured the RFP level the next day.

Consecutive SoxS fusion chain

To further enhance the CRISPRa efficiency in bacteria, we designed a consecutive C-to N-terminal fusion domain of SoxS that was thought to enable synergistic effects and increases the target flexibility alongside (elimination of phase dependency).

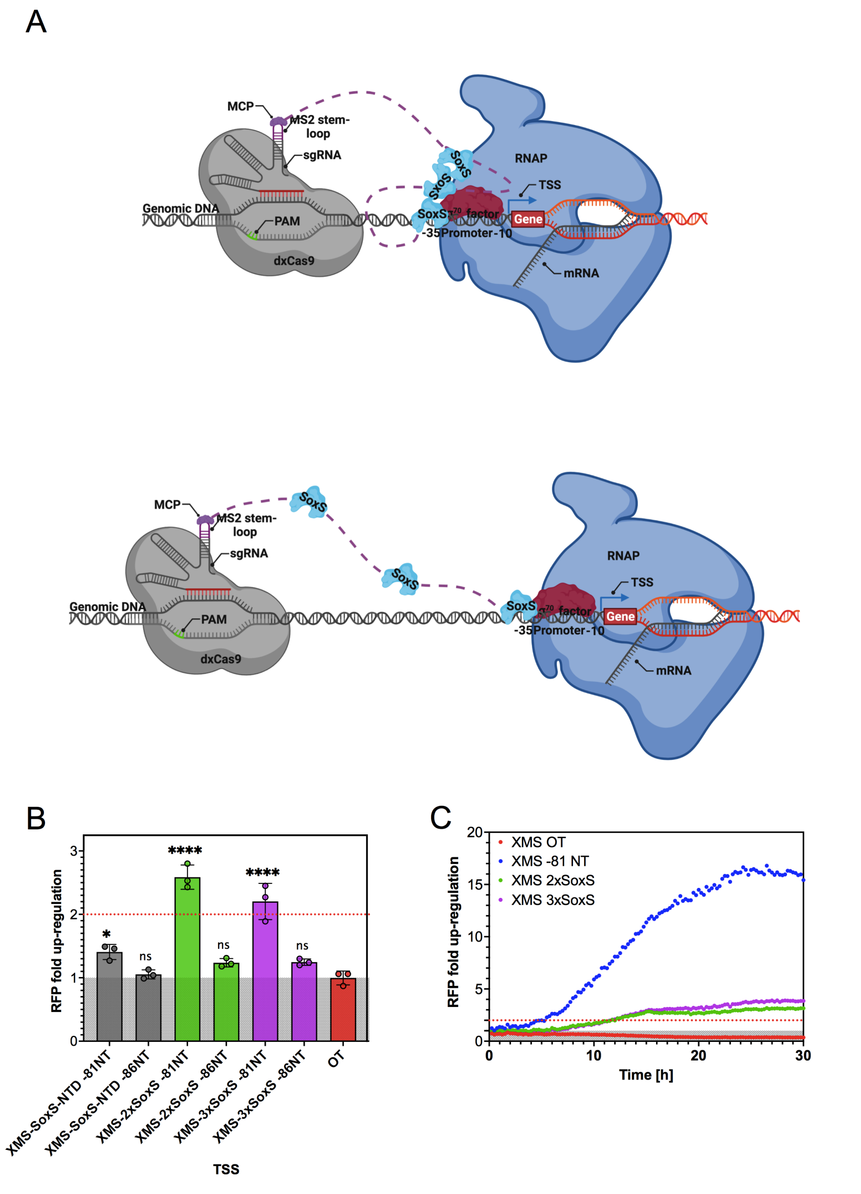

Figure S2: Consecutive C-to N-terminal SoxS fusion chain (**A**) The SoxS fusion chain was supposed to enhance the CRISPRa efficiency and to increase the CRISPRa flexibility and thus should enable up-regulation form target sites outside the up-regulative window, or out of phase respectively. (**B**) A drop of up-regulated RFP signal was visible rather than the expected increase upon up-regulation with the consecutive SoxS chain. Afterwards, we designed a single N-terminal SoxS fusion construct, and observed an almost entirely erased up-regulative propensity when targeting position -81NT. We conclude that the NTD of SoxS is essential for its activator function. (**C**) Continuous monitoring of RFP up-regulation by consecutive SoxS chain supports the observations in plate-reader experiments. Results of all statistical analyses are provided in the Supplementary File Statistics. Asterisk **** = $P < 0{,}0001$. OT= off-target control, XMS -81NT= native XMS construct used as positive control

Continuous monitoring of low-performing non-canonical PAM sites

To verify the functionality of SMS at non-canonical PAMs we selected 4 PAM sequences that lead to rather low up-regulation levels compared to other PAM sites, and conducted continuous monitoring.

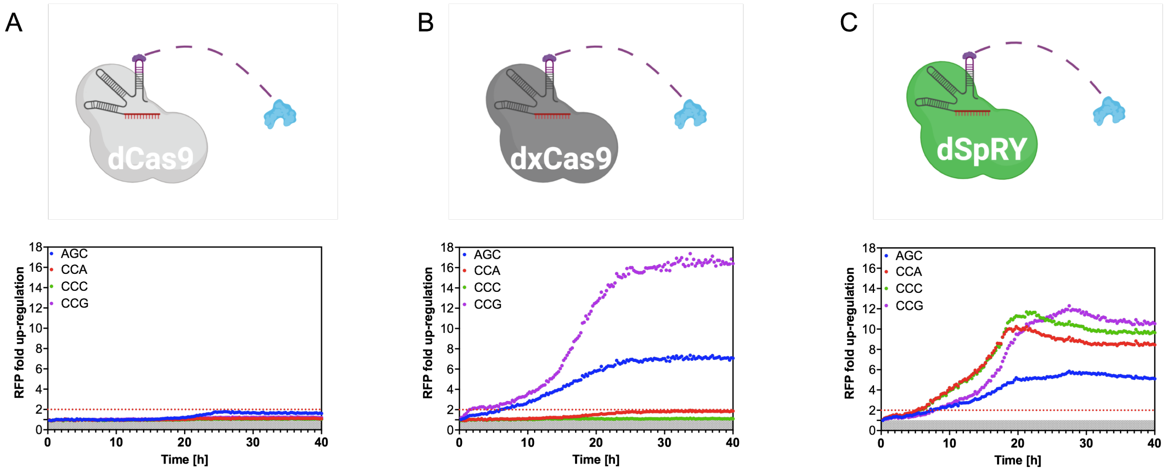

Figure S3: BioLector microfermentation verifies PAM independency of SMS. Since the level of RFP up-regulation is time dependent and can thus be readily misjudged, we selected 4 PAM sequences (AGC, CCA, CCC, CCG) with rather low fold change in RFP up-regulation by SMS. We monitored the CRISPRa constructs again for these PAMs in BioLector microfermentation systems for 40 h. (**A**) CMS monitoring of AGC, CCA, CCG and CCC PAMs did not result in any considerable up-regulation above the threshold. (**B**) XMS showed stronger up-regulation as SMS at CCG PAM (17-fold), which was also considerable higher in plate-reader studies and similar up-regulation for AGC PAM (7-fold), but no up-regulation at CCA and CCC PAM. (**C**) SMS experiments resulted in decisive up-regulation at all 4 PAMs (AGC 6-fold, CCA 10-fold, CCC and CCG 12-fold).

Determination of optimal CRISPRa target site

Since CRISPRa does not follow simple distance metrics, we determinded the optimal target site for our endogenous model protein ECS, via an endogenous ECS promoter driven RFP on a plasmid and screened position -73 to -82 on the non-template strand.

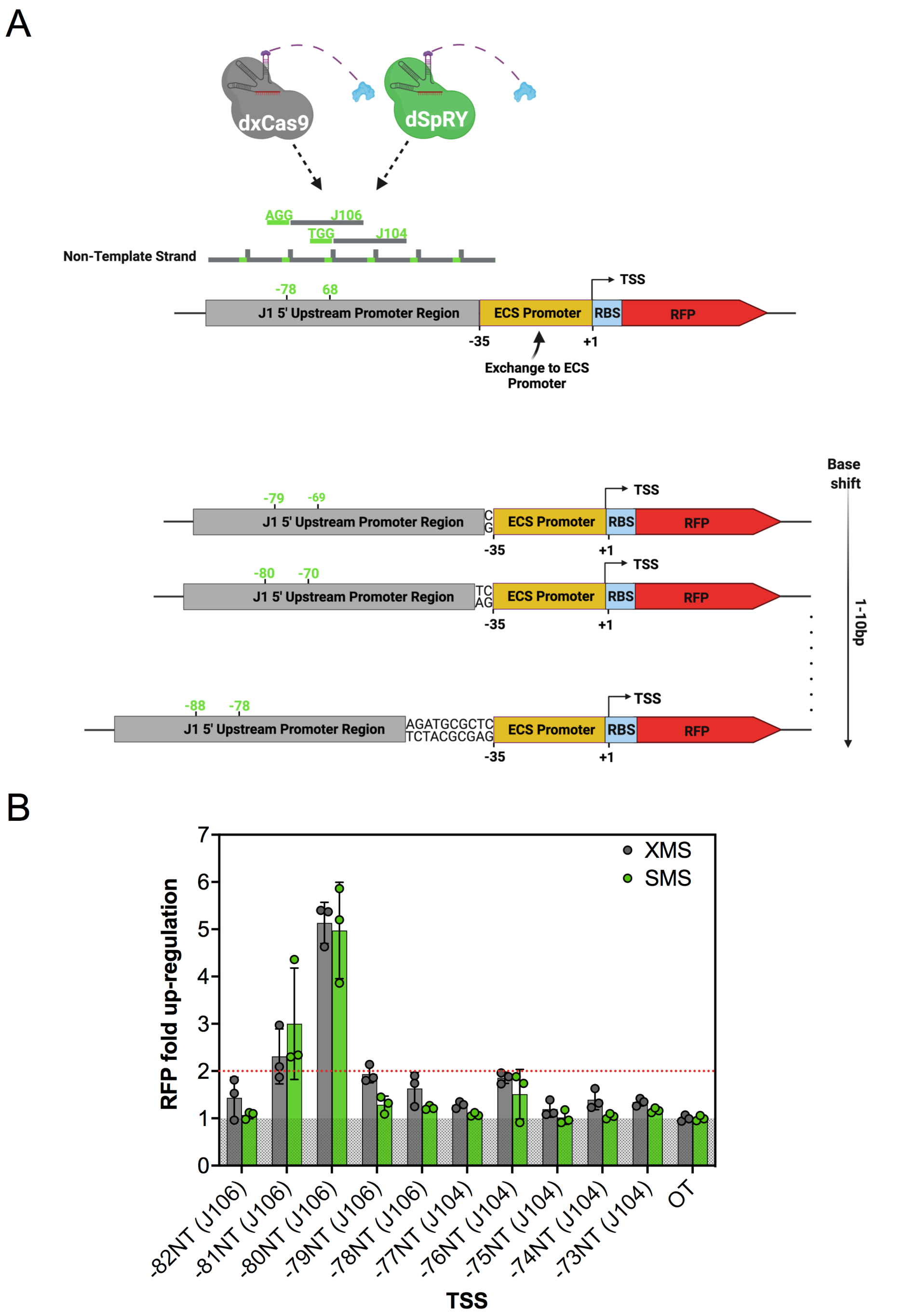

Figure S4 Determination of optimal CRISPRa target site using a plasmid-based endogenous promoter. **(A)** Exchange of synthetic to endogenous *ECS* promoter in order to decipher the optimal target site for subsequent endogenous up-regulation of *ECS*. We incorporated 1 to 10 additional base pairs upstream the promoter. Employing scRNA J104 and J106 thereby allowed us to screen position -73 to -82 at NT strand. (**B**) Since both scRNAs are adjacent to canonical PAMs in this setup, we used XMS and SMS for screening, whereby both of them revealed a peak in RFP up-regulation at position -80 NT. OT= off-target control

SMS dependent endogenous gene up-regulation at non-canonical PAM sites

Although CRISPRa do not follow simple distance metrics (1), we assumed that activation for most endogenous genes might be somewhere around position -80NT. Therefore we chose two further chaperons, *surA* and *fkpa* for endogenous up-regulation and used *E. coli* production strain BL21(DE3) as a testing host. *poxB* was used as a positive control targeted on a canonical PAM. Results of statistical calculations can be found in the Supplementary File Statistics.

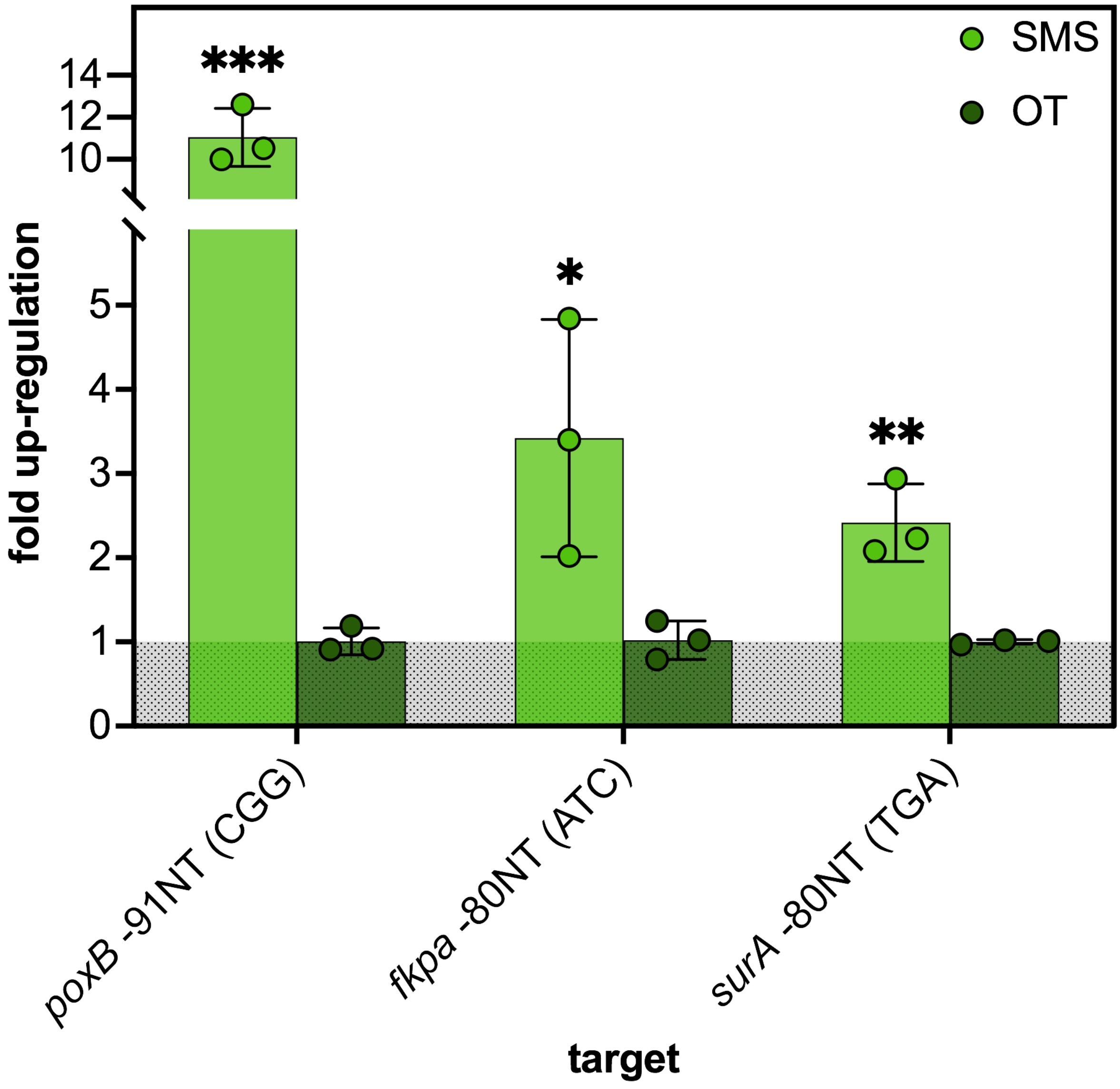

Figure S5 SMS mediated up-regulation at non-canonical PAM sites of *surA* and *fkpa* in *E. coli* BL21(DE3) production strains. Results of statistical analyses are provided in the Supplementary File Statistics. Asterisk *** = *P* < 0,001, Asterisk ** = *P* < 0,01, Asterisk * = *P* ≤ 0,05. OT= off-target control

Schematic overview of the design for simultaneous CRISPRa multi-gene expression control

A

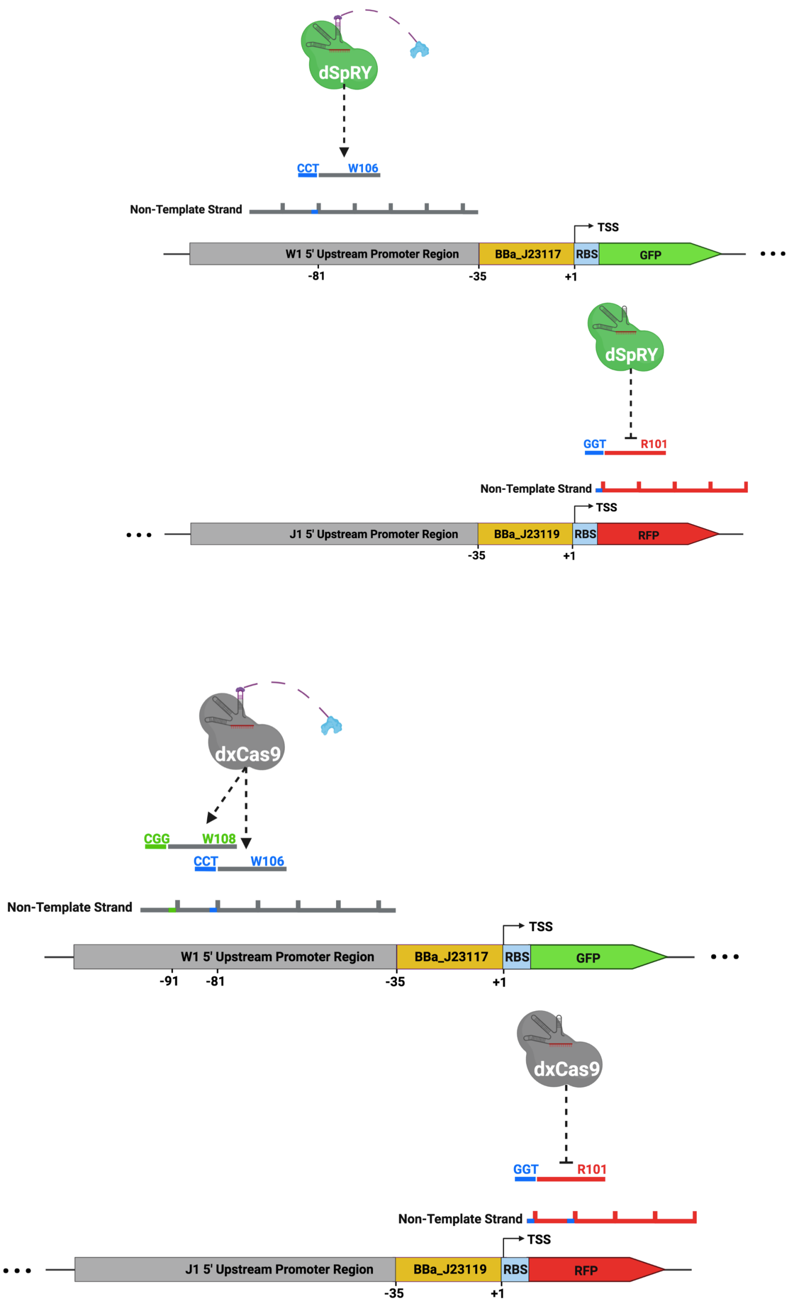

Figure S6: (**A**) SMS was used to target non-canonical CCT PAM via scRNA W106 for GFP up-regulation and non-canonical GGT PAM via gRNA R101 for RFP down-regulation. Both, scRNA and gRNA, have been expressed on the same CRISPRa-SMS plasmid construct and were competing for the interaction with dSpRY. (**B**) The same experiment has been conducted for XMS. However, since XMS is not functional at non-canonical CCT for GFP up-regulation, we verified its general functionality by targeting position -91NT which is adjacent to a canonical CGG PAM.

Table S1 List of RFP/GFP target promoter sequences

Target promoter	DNA sequence
BBa_J23107	TTGACAGCTAGCTCAGTCCTAGGGATTGTGCTAGC
*ESC*_Promoter	ATTAATTGACGCTAAAGTCAGTAAAGTTAATC
BBa_J23108	CTGACAGCTAGCTCAGTCCTAGGTATAATGCTAGC
BBa_J23100	TTGACGGCTAGCTCAGTCCTAGGTACAGTGCTAGC

Table S2 List of scRNA target DNA sequences

scRNA	target	base pairing sequence	PAM	scRNA backbone
J104	J1-pJF076Sa	TGGAACCCAAAGGACGCCTT	TGG	GTTTTAGAG<br>CTAGAAATA<br>GCAAGTTAA<br>AATAAGGCT<br>AGTCCGTTA<br>TCAACTTGA<br>AAAAGTGGC<br>ACATGAGGA<br>TCACCCATG<br>TGCTTTTTT
J106	J1-pJF076Sa	AGGACGCCTTTGGTAACCGC	AGG	
J107	J1-pJF076Sa	CGGTGTCCTGCGGTTACCAA	AGG	
J108	J1-pJF076Sa	TGGTAACCGCAGGACACCGC	AGG	
J109	J1-pJF076Sa	AGGTATCCTGCGGTGTCCTG	CGG	
J111	J1-pJF076Sa	GGGCGACCTCAGGTATCCTG	CGG	
P108	Endogenous *poxB*	CCCGATGAAAGGAATATCAT	CGG	
E106	Endogenous *ECS*	AAAAAACACTTTTGCGCCAA	TTA	
F106	Endogenous *fkpa*	GGGCAGCAAAGTTAAGTAGA	ATC	
S106	Endogenous *surA*	AGTGGTGTTAGGCACGGCAT	TGA	
W108	pCD002	GAAGATCCGGCCTGCAGCCA	AGG	
W106	pCD002	CCGGATCTTCCACAACACGC	CCT	

Table S3 List of gRNA target sequence

gRNA	target	base pairing sequence	PAM	gRNA backbone
R101	RFP-pJF076Sa	ATGGCGAGTAGCGAAGACGT	GGT	GTTTTAGAG
R102	RFP-pJF076Sa	CGAAGACGTTATCAAAGAGT	CTA	CTAGAAATA<br>GCAAGTTAA<br>AATAAGGCT<br>AGTCCGTTA<br>TCAACTTGA<br>AAAAGTGGC<br>ACCGAGTCG<br>GTGCTTTTT<br>T

Table S4 List of RT-qPCR primers

target	strand	DNA sequence
poxB	Fwd	GGTCTTAGTGACAGTCTTAATC
poxB	Rev	GGAATATGAGCGGCAATC
ECS	Fwd	GCCAGCGATACCTTCGATAAA
ECS	Rev	CGGCGTCAGGATGTTGTAAA
fkpa	Fwd	CGATCAGCGTACCTTTGTAGTT
fkpa	Rev	CCTCTTCAACTGGTCTGGTTTAT
surA	Fwd	CCAGCCGAATGAAGAGTGAA
surA	Rev	GCTGGGCTACACCAGATATT

Table S5 List of dual plasmid combinations for Figure 2ATable S5 List of dual plasmid combinations for Figure 2A and B

CRISPRa Plasmid	Cas9 variant	RFP Plasmid	J1 insertion	RFP Promoter	scRNA	distance to TSS	strand
pCD565	dxCas9	pJF076Sa		BBa_J23107	J104	-71	NT
pCD565	dxCas9	pJF076Sa	C	BBa_J23107	J107	-71	T
pCD565	dxCas9	pJF076Sa	CGCTC	BBa_J23107	J104	-76	NT
pCD565	dxCas9	pJF076Sa	GCGCTC	BBa_J23107	J107	-76	T
pCD565	dxCas9	pJF076Sa		BBa_J23107	J106	-81	NT
pCD565	dxCas9	pJF076Sa	C	BBa_J23107	J109	-81	T
pCD565	dxCas9	pJF076Sa	CGCTC	BBa_J23107	J106	-86	NT
pCD565	dxCas9	pJF076Sa	GCGCTC	BBa_J23107	J109	-86	T
pCD565	dxCas9	pJF076Sa		BBa_J23107	J108	-91	NT
pCD565	dxCas9	pJF076Sa	C	BBa_J23107	J111	-91	T
pCD565	dxCas9	pJF076Sa	CGCTC	BBa_J23107	J108	-96	NT
pCD565	dxCas9	pJF076Sa	GCGCTC	BBa_J23107	J111	-96	T
pCD565	dxCas9	pJF076Sa		BBa_J23107	J306	OT	
pCD565	dSpRY	pJF076Sa		BBa_J23107	J104	-71	NT
pCD565	dSpRY	pJF076Sa	C	BBa_J23107	J107	-71	T
pCD565	dSpRY	pJF076Sa	CGCTC	BBa_J23107	J104	-76	NT
pCD565	dSpRY	pJF076Sa	GCGCTC	BBa_J23107	J107	-76	T
pCD565	dSpRY	pJF076Sa		BBa_J23107	J106	-81	NT
pCD565	dSpRY	pJF076Sa	C	BBa_J23107	J109	-81	T
pCD565	dSpRY	pJF076Sa	CGCTC	BBa_J23107	J106	-86	NT
pCD565	dSpRY	pJF076Sa	GCGCTC	BBa_J23107	J109	-86	T
pCD565	dSpRY	pJF076Sa		BBa_J23107	J108	-91	NT
pCD565	dSpRY	pJF076Sa	C	BBa_J23107	J111	-91	T

pCD565	dSpRY	pJF076Sa	CGCTC	BBa_J23107	J108	-96	NT
pCD565	dSpRY	pJF076Sa	GCGCTC	BBa_J23107	J111	-96	T
pCD565	dSpRY	pJF076Sa		BBa_J23107	J306	OT	

Table S6 List of dual plasmid combinations for Figure 2D

CRISPRa Plasmid	Cas9 variant	RFP Plasmid	PAM site	RFP Promoter	scRNA	distance to TSS	strand
pCD565	dCas9	pJF076Sa	AAA	BBa_J23107	J106	-81	NT
pCD565	dCas9	pJF076Sa	AAC	BBa_J23107	J106	-81	NT
pCD565	dCas9	pJF076Sa	AAG	BBa_J23107	J106	-81	NT
pCD565	dCas9	pJF076Sa	AAT	BBa_J23107	J106	-81	NT
pCD565	dCas9	pJF076Sa	ACA	BBa_J23107	J106	-81	NT
pCD565	dCas9	pJF076Sa	ACC	BBa_J23107	J106	-81	NT
pCD565	dCas9	pJF076Sa	ACG	BBa_J23107	J106	-81	NT
pCD565	dCas9	pJF076Sa	ACT	BBa_J23107	J106	-81	NT
pCD565	dCas9	pJF076Sa	AGA	BBa_J23107	J106	-81	NT
pCD565	dCas9	pJF076Sa	AGC	BBa_J23107	J106	-81	NT
pCD565	dCas9	pJF076Sa	AGG	BBa_J23107	J106	-81	NT
pCD565	dCas9	pJF076Sa	AGT	BBa_J23107	J106	-81	NT
pCD565	dCas9	pJF076Sa	ATA	BBa_J23107	J106	-81	NT
pCD565	dCas9	pJF076Sa	ATC	BBa_J23107	J106	-81	NT
pCD565	dCas9	pJF076Sa	ATG	BBa_J23107	J106	-81	NT
pCD565	dCas9	pJF076Sa	ATT	BBa_J23107	J106	-81	NT
pCD565	dCas9	pJF076Sa	CAA	BBa_J23107	J106	-81	NT
pCD565	dCas9	pJF076Sa	CAC	BBa_J23107	J106	-81	NT
pCD565	dCas9	pJF076Sa	CAG	BBa_J23107	J106	-81	NT
pCD565	dCas9	pJF076Sa	CAT	BBa_J23107	J106	-81	NT
pCD565	dCas9	pJF076Sa	CCA	BBa_J23107	J106	-81	NT
pCD565	dCas9	pJF076Sa	CCC	BBa_J23107	J106	-81	NT
pCD565	dCas9	pJF076Sa	CCG	BBa_J23107	J106	-81	NT
pCD565	dCas9	pJF076Sa	CCT	BBa_J23107	J106	-81	NT
pCD565	dCas9	pJF076Sa	CGA	BBa_J23107	J106	-81	NT
pCD565	dCas9	pJF076Sa	CGC	BBa_J23107	J106	-81	NT
pCD565	dCas9	pJF076Sa	CGG	BBa_J23107	J106	-81	NT
pCD565	dCas9	pJF076Sa	CGT	BBa_J23107	J106	-81	NT
pCD565	dCas9	pJF076Sa	CTA	BBa_J23107	J106	-81	NT
pCD565	dCas9	pJF076Sa	CTC	BBa_J23107	J106	-81	NT
pCD565	dCas9	pJF076Sa	CTG	BBa_J23107	J106	-81	NT
pCD565	dCas9	pJF076Sa	CTT	BBa_J23107	J106	-81	NT
pCD565	dCas9	pJF076Sa	GAA	BBa_J23107	J106	-81	NT
pCD565	dCas9	pJF076Sa	GAC	BBa_J23107	J106	-81	NT
pCD565	dCas9	pJF076Sa	GAG	BBa_J23107	J106	-81	NT

pCD565	dCas9	pJF076Sa	GAT	BBa_J23107	J106	-81	NT
pCD565	dCas9	pJF076Sa	GCA	BBa_J23107	J106	-81	NT
pCD565	dCas9	pJF076Sa	GCC	BBa_J23107	J106	-81	NT
pCD565	dCas9	pJF076Sa	GCG	BBa_J23107	J106	-81	NT
pCD565	dCas9	pJF076Sa	GCT	BBa_J23107	J106	-81	NT
pCD565	dCas9	pJF076Sa	GGA	BBa_J23107	J106	-81	NT
pCD565	dCas9	pJF076Sa	GGC	BBa_J23107	J106	-81	NT
pCD565	dCas9	pJF076Sa	GGG	BBa_J23107	J106	-81	NT
pCD565	dCas9	pJF076Sa	GGT	BBa_J23107	J106	-81	NT
pCD565	dCas9	pJF076Sa	GTA	BBa_J23107	J106	-81	NT
pCD565	dCas9	pJF076Sa	GTC	BBa_J23107	J106	-81	NT
pCD565	dCas9	pJF076Sa	GTG	BBa_J23107	J106	-81	NT
pCD565	dCas9	pJF076Sa	GTT	BBa_J23107	J106	-81	NT
pCD565	dCas9	pJF076Sa	TAA	BBa_J23107	J106	-81	NT
pCD565	dCas9	pJF076Sa	TAC	BBa_J23107	J106	-81	NT
pCD565	dCas9	pJF076Sa	TAG	BBa_J23107	J106	-81	NT
pCD565	dCas9	pJF076Sa	TAT	BBa_J23107	J106	-81	NT
pCD565	dCas9	pJF076Sa	TCA	BBa_J23107	J106	-81	NT
pCD565	dCas9	pJF076Sa	TCC	BBa_J23107	J106	-81	NT
pCD565	dCas9	pJF076Sa	TCG	BBa_J23107	J106	-81	NT
pCD565	dCas9	pJF076Sa	TCT	BBa_J23107	J106	-81	NT
pCD565	dCas9	pJF076Sa	TGA	BBa_J23107	J106	-81	NT
pCD565	dCas9	pJF076Sa	TGC	BBa_J23107	J106	-81	NT
pCD565	dCas9	pJF076Sa	TGG	BBa_J23107	J106	-81	NT
pCD565	dCas9	pJF076Sa	TGT	BBa_J23107	J106	-81	NT
pCD565	dCas9	pJF076Sa	TTA	BBa_J23107	J106	-81	NT
pCD565	dCas9	pJF076Sa	TTC	BBa_J23107	J106	-81	NT
pCD565	dCas9	pJF076Sa	TTG	BBa_J23107	J106	-81	NT
pCD565	dCas9	pJF076Sa	TTT	BBa_J23107	J106	-81	NT
pCD565	dCas9	pJF076Sa	OT	BBa_J23107	J106	-81	NT
pCD565	dxCas9	pJF076Sa	AAA	BBa_J23107	J106	-81	NT
pCD565	dxCas9	pJF076Sa	AAC	BBa_J23107	J106	-81	NT
pCD565	dxCas9	pJF076Sa	AAG	BBa_J23107	J106	-81	NT
pCD565	dxCas9	pJF076Sa	AAT	BBa_J23107	J106	-81	NT
pCD565	dxCas9	pJF076Sa	ACA	BBa_J23107	J106	-81	NT
pCD565	dxCas9	pJF076Sa	ACC	BBa_J23107	J106	-81	NT
pCD565	dxCas9	pJF076Sa	ACG	BBa_J23107	J106	-81	NT
pCD565	dxCas9	pJF076Sa	ACT	BBa_J23107	J106	-81	NT

pCD565	dxCas9	pJF076Sa	AGA	BBa_J23107	J106	-81	NT
pCD565	dxCas9	pJF076Sa	AGC	BBa_J23107	J106	-81	NT
pCD565	dxCas9	pJF076Sa	AGG	BBa_J23107	J106	-81	NT
pCD565	dxCas9	pJF076Sa	AGT	BBa_J23107	J106	-81	NT
pCD565	dxCas9	pJF076Sa	ATA	BBa_J23107	J106	-81	NT
pCD565	dxCas9	pJF076Sa	ATC	BBa_J23107	J106	-81	NT
pCD565	dxCas9	pJF076Sa	ATG	BBa_J23107	J106	-81	NT
pCD565	dxCas9	pJF076Sa	ATT	BBa_J23107	J106	-81	NT
pCD565	dxCas9	pJF076Sa	CAA	BBa_J23107	J106	-81	NT
pCD565	dxCas9	pJF076Sa	CAC	BBa_J23107	J106	-81	NT
pCD565	dxCas9	pJF076Sa	CAG	BBa_J23107	J106	-81	NT
pCD565	dxCas9	pJF076Sa	CAT	BBa_J23107	J106	-81	NT
pCD565	dxCas9	pJF076Sa	CCA	BBa_J23107	J106	-81	NT
pCD565	dxCas9	pJF076Sa	CCC	BBa_J23107	J106	-81	NT
pCD565	dxCas9	pJF076Sa	CCG	BBa_J23107	J106	-81	NT
pCD565	dxCas9	pJF076Sa	CCT	BBa_J23107	J106	-81	NT
pCD565	dxCas9	pJF076Sa	CGA	BBa_J23107	J106	-81	NT
pCD565	dxCas9	pJF076Sa	CGC	BBa_J23107	J106	-81	NT
pCD565	dxCas9	pJF076Sa	CGG	BBa_J23107	J106	-81	NT
pCD565	dxCas9	pJF076Sa	CGT	BBa_J23107	J106	-81	NT
pCD565	dxCas9	pJF076Sa	CTA	BBa_J23107	J106	-81	NT
pCD565	dxCas9	pJF076Sa	CTC	BBa_J23107	J106	-81	NT
pCD565	dxCas9	pJF076Sa	CTG	BBa_J23107	J106	-81	NT
pCD565	dxCas9	pJF076Sa	CTT	BBa_J23107	J106	-81	NT
pCD565	dxCas9	pJF076Sa	GAA	BBa_J23107	J106	-81	NT
pCD565	dxCas9	pJF076Sa	GAC	BBa_J23107	J106	-81	NT
pCD565	dxCas9	pJF076Sa	GAG	BBa_J23107	J106	-81	NT
pCD565	dxCas9	pJF076Sa	GAT	BBa_J23107	J106	-81	NT
pCD565	dxCas9	pJF076Sa	GCA	BBa_J23107	J106	-81	NT
pCD565	dxCas9	pJF076Sa	GCC	BBa_J23107	J106	-81	NT
pCD565	dxCas9	pJF076Sa	GCG	BBa_J23107	J106	-81	NT
pCD565	dxCas9	pJF076Sa	GCT	BBa_J23107	J106	-81	NT
pCD565	dxCas9	pJF076Sa	GGA	BBa_J23107	J106	-81	NT
pCD565	dxCas9	pJF076Sa	GGC	BBa_J23107	J106	-81	NT
pCD565	dxCas9	pJF076Sa	GGG	BBa_J23107	J106	-81	NT
pCD565	dxCas9	pJF076Sa	GGT	BBa_J23107	J106	-81	NT
pCD565	dxCas9	pJF076Sa	GTA	BBa_J23107	J106	-81	NT
pCD565	dxCas9	pJF076Sa	GTC	BBa_J23107	J106	-81	NT

pCD565	dxCas9	pJF076Sa	GTG	BBa_J23107	J106	-81	NT
pCD565	dxCas9	pJF076Sa	GTT	BBa_J23107	J106	-81	NT
pCD565	dxCas9	pJF076Sa	TAA	BBa_J23107	J106	-81	NT
pCD565	dxCas9	pJF076Sa	TAC	BBa_J23107	J106	-81	NT
pCD565	dxCas9	pJF076Sa	TAG	BBa_J23107	J106	-81	NT
pCD565	dxCas9	pJF076Sa	TAT	BBa_J23107	J106	-81	NT
pCD565	dxCas9	pJF076Sa	TCA	BBa_J23107	J106	-81	NT
pCD565	dxCas9	pJF076Sa	TCC	BBa_J23107	J106	-81	NT
pCD565	dxCas9	pJF076Sa	TCG	BBa_J23107	J106	-81	NT
pCD565	dxCas9	pJF076Sa	TCT	BBa_J23107	J106	-81	NT
pCD565	dxCas9	pJF076Sa	TGA	BBa_J23107	J106	-81	NT
pCD565	dxCas9	pJF076Sa	TGC	BBa_J23107	J106	-81	NT
pCD565	dxCas9	pJF076Sa	TGG	BBa_J23107	J106	-81	NT
pCD565	dxCas9	pJF076Sa	TGT	BBa_J23107	J106	-81	NT
pCD565	dxCas9	pJF076Sa	TTA	BBa_J23107	J106	-81	NT
pCD565	dxCas9	pJF076Sa	TTC	BBa_J23107	J106	-81	NT
pCD565	dxCas9	pJF076Sa	TTG	BBa_J23107	J106	-81	NT
pCD565	dxCas9	pJF076Sa	TTT	BBa_J23107	J106	-81	NT
pCD565	dxCas9	pJF076Sa	OT	BBa_J23107	J106	-81	NT
pCD565	dSpRY	pJF076Sa	AAA	BBa_J23107	J106	-81	NT
pCD565	dSpRY	pJF076Sa	AAC	BBa_J23107	J106	-81	NT
pCD565	dSpRY	pJF076Sa	AAG	BBa_J23107	J106	-81	NT
pCD565	dSpRY	pJF076Sa	AAT	BBa_J23107	J106	-81	NT
pCD565	dSpRY	pJF076Sa	ACA	BBa_J23107	J106	-81	NT
pCD565	dSpRY	pJF076Sa	ACC	BBa_J23107	J106	-81	NT
pCD565	dSpRY	pJF076Sa	ACG	BBa_J23107	J106	-81	NT
pCD565	dSpRY	pJF076Sa	ACT	BBa_J23107	J106	-81	NT
pCD565	dSpRY	pJF076Sa	AGA	BBa_J23107	J106	-81	NT
pCD565	dSpRY	pJF076Sa	AGC	BBa_J23107	J106	-81	NT
pCD565	dSpRY	pJF076Sa	AGG	BBa_J23107	J106	-81	NT
pCD565	dSpRY	pJF076Sa	AGT	BBa_J23107	J106	-81	NT
pCD565	dSpRY	pJF076Sa	ATA	BBa_J23107	J106	-81	NT
pCD565	dSpRY	pJF076Sa	ATC	BBa_J23107	J106	-81	NT
pCD565	dSpRY	pJF076Sa	ATG	BBa_J23107	J106	-81	NT
pCD565	dSpRY	pJF076Sa	ATT	BBa_J23107	J106	-81	NT
pCD565	dSpRY	pJF076Sa	CAA	BBa_J23107	J106	-81	NT
pCD565	dSpRY	pJF076Sa	CAC	BBa_J23107	J106	-81	NT
pCD565	dSpRY	pJF076Sa	CAG	BBa_J23107	J106	-81	NT

pCD565	dSpRY	pJF076Sa	CAT	BBa_J23107	J106	-81	NT
pCD565	dSpRY	pJF076Sa	CCA	BBa_J23107	J106	-81	NT
pCD565	dSpRY	pJF076Sa	CCC	BBa_J23107	J106	-81	NT
pCD565	dSpRY	pJF076Sa	CCG	BBa_J23107	J106	-81	NT
pCD565	dSpRY	pJF076Sa	CCT	BBa_J23107	J106	-81	NT
pCD565	dSpRY	pJF076Sa	CGA	BBa_J23107	J106	-81	NT
pCD565	dSpRY	pJF076Sa	CGC	BBa_J23107	J106	-81	NT
pCD565	dSpRY	pJF076Sa	CGG	BBa_J23107	J106	-81	NT
pCD565	dSpRY	pJF076Sa	CGT	BBa_J23107	J106	-81	NT
pCD565	dSpRY	pJF076Sa	CTA	BBa_J23107	J106	-81	NT
pCD565	dSpRY	pJF076Sa	CTC	BBa_J23107	J106	-81	NT
pCD565	dSpRY	pJF076Sa	CTG	BBa_J23107	J106	-81	NT
pCD565	dSpRY	pJF076Sa	CTT	BBa_J23107	J106	-81	NT
pCD565	dSpRY	pJF076Sa	GAA	BBa_J23107	J106	-81	NT
pCD565	dSpRY	pJF076Sa	GAC	BBa_J23107	J106	-81	NT
pCD565	dSpRY	pJF076Sa	GAG	BBa_J23107	J106	-81	NT
pCD565	dSpRY	pJF076Sa	GAT	BBa_J23107	J106	-81	NT
pCD565	dSpRY	pJF076Sa	GCA	BBa_J23107	J106	-81	NT
pCD565	dSpRY	pJF076Sa	GCC	BBa_J23107	J106	-81	NT
pCD565	dSpRY	pJF076Sa	GCG	BBa_J23107	J106	-81	NT
pCD565	dSpRY	pJF076Sa	GCT	BBa_J23107	J106	-81	NT
pCD565	dSpRY	pJF076Sa	GGA	BBa_J23107	J106	-81	NT
pCD565	dSpRY	pJF076Sa	GGC	BBa_J23107	J106	-81	NT
pCD565	dSpRY	pJF076Sa	GGG	BBa_J23107	J106	-81	NT
pCD565	dSpRY	pJF076Sa	GGT	BBa_J23107	J106	-81	NT
pCD565	dSpRY	pJF076Sa	GTA	BBa_J23107	J106	-81	NT
pCD565	dSpRY	pJF076Sa	GTC	BBa_J23107	J106	-81	NT
pCD565	dSpRY	pJF076Sa	GTG	BBa_J23107	J106	-81	NT
pCD565	dSpRY	pJF076Sa	GTT	BBa_J23107	J106	-81	NT
pCD565	dSpRY	pJF076Sa	TAA	BBa_J23107	J106	-81	NT
pCD565	dSpRY	pJF076Sa	TAC	BBa_J23107	J106	-81	NT
pCD565	dSpRY	pJF076Sa	TAG	BBa_J23107	J106	-81	NT
pCD565	dSpRY	pJF076Sa	TAT	BBa_J23107	J106	-81	NT
pCD565	dSpRY	pJF076Sa	TCA	BBa_J23107	J106	-81	NT
pCD565	dSpRY	pJF076Sa	TCC	BBa_J23107	J106	-81	NT
pCD565	dSpRY	pJF076Sa	TCG	BBa_J23107	J106	-81	NT
pCD565	dSpRY	pJF076Sa	TCT	BBa_J23107	J106	-81	NT
pCD565	dSpRY	pJF076Sa	TGA	BBa_J23107	J106	-81	NT

pCD565	dSpRY	pJF076Sa	TGC	BBa_J23107	J106	-81	NT
pCD565	dSpRY	pJF076Sa	TGG	BBa_J23107	J106	-81	NT
pCD565	dSpRY	pJF076Sa	TGT	BBa_J23107	J106	-81	NT
pCD565	dSpRY	pJF076Sa	TTA	BBa_J23107	J106	-81	NT
pCD565	dSpRY	pJF076Sa	TTC	BBa_J23107	J106	-81	NT
pCD565	dSpRY	pJF076Sa	TTG	BBa_J23107	J106	-81	NT
pCD565	dSpRY	pJF076Sa	TTT	BBa_J23107	J106	-81	NT
pCD565	dSpRY	pJF076Sa	OT	BBa_J23107	J106	-81	NT

Table S7 List of dual plasmid combinations for Figure 3B

CRISPRa Plasmid	Cas9 variant	GFP/RFP Plasmid	Promoter	scRNA	distance to TSS	strand
pCD565	dxCas9	pCD002	J23107	W106	-81	NT
pCD565	dSpRY	pCD002	J23107	W106	-81	NT
pCD565	dxCas9	integrated	J23107	W106	-81	NT
pCD565	dSpRY	integrated	J23107	W106	-81	NT

Table S8 List of dual plasmid combinations for RFP down-regulation in Figure 4B, C and E

CRISPRa Plasmid	Cas9 variant	RFP Plasmid	RFP Promoter	gRNA	distance to TSS	strand
pCD565	dSpRY	pJF076Sa	J23119	R101	+27	NT
pCD565	dSpRY	pJF076Sa	J23119	R102	+38	NT
pCD565	dSpRY	pJF076Sa	J23119	J306		OT

Table S9 List of dual plasmid combinations for simultaneous up-and down-regulation in Figure 4C and E

CRISPRa Plasmid	Cas9 variant	RFP/GFP Plasmid	RFP/GFP Promoter	gRNA scRNA	distance to TSS	strand
pCD565	dSpRY	pJF076Sa	J23119<br>J23107	R101<br>W106	+27<br>-81	NT<br>NT
pCD565	dxCas9	pJF076Sa	J23119<br>J23107	R101<br>W106	+27<br>-81	NT<br>NT
pCD565	dxCas9	pJF076Sa	J23107	W106	-91	NT
pCD565	dSpRY	pJF076Sa	J23119	J306		OT
pCD565	dxCas9	pJF076Sa	J23119	J306		OT

Table S10 List of dual plasmid combinations for Figure S2

CRISPRa Plasmid	Cas9 variant	RFP Plasmid	RFP Promoter	scRNA	distance to TSS	strand
pCD565 SoxS-CTD	dxCas9	pJF076Sa	J23119	J106	-81	NT
pCD565 2xSoxS	dxCas9	pJF076Sa	J23119	J106	-81	NT
pCD565 3xSoxS	dxCas9	pJF076Sa	J23119	J106	-81	NT
pCD565	dxCas9	pJF076Sa	J23119	J306		OT

Table S11 List of dual plasmid combinations for Figure S5B

CRISPRa Plasmid	Cas9 variant	RFP Plasmid	J1 insertion	RFP Promoter	scRNA	distance to TSS	strand
pCD565	dxCas9	pJF076Sa	GCGCTC	*endogenous ECS*	J104	-74	NT
pCD565	dxCas9	pJF076Sa	TGCGCTC	endogenous *ECS*	J104	-75	NT
pCD565	dxCas9	pJF076Sa	ATGCGCTC	endogenous *ECS*	J104	-76	NT
pCD565	dxCas9	pJF076Sa	GATGCGCTC	endogenous *ECS*	J104	-77	NT
pCD565	dxCas9	pJF076Sa		endogenous *ECS*	J106	-78	NT
pCD565	dxCas9	pJF076Sa	C	endogenous *ECS*	J106	-79	NT
pCD565	dxCas9	pJF076Sa	TC	endogenous *ECS*	J106	-80	NT
pCD565	dxCas9	pJF076Sa	CTC	endogenous *ECS*	J106	-81	NT
pCD565	dxCas9	pJF076Sa	GCTC	endogenous *ECS*	J106	-82	NT
pCD565	dxCas9	pJF076Sa		endogenous *ECS*	J306	OT	
pCD565	dSpRY	pJF076Sa	CGCTC	endogenous *ECS*	J104	-73	NT
pCD565	dSpRY	pJF076Sa	GCGCTC	endogenous *ECS*	J104	-74	NT
pCD565	dSpRY	pJF076Sa	TGCGCTC	endogenous *ECS*	J104	-75	NT
pCD565	dSpRY	pJF076Sa	ATGCGCTC	endogenous *ECS*	J104	-76	NT
pCD565	dSpRY	pJF076Sa	GATGCGCTC	endogenous *ECS*	J104	-77	NT
pCD565	dSpRY	pJF076Sa		endogenous *ECS*	J106	-78	NT
pCD565	dSpRY	pJF076Sa	C	endogenous *ECS*	J106	-79	NT

pCD565	dSpRY	pJF076Sa	TC	endogenous *ECS*	J106	-80	NT
pCD565	dSpRY	pJF076Sa	CTC	endogenous *ECS*	J106	-81	NT
pCD565	dSpRY	pJF076Sa	GCTC	endogenous *ECS*	J106	-82	NT
pCD565	dSpRY	pJF076Sa		endogenous *ECS*	J306	OT	

***E. coli* codon optimized nucleic acid sequence of dSpRY:**

ATGGACAAAAAGTATTCGATCGGATTAGCTATCGGTACCAATAGCGTTGGGTGG
GCGGTCATTACAGACGAGTATAAGGTACCGAGCAAGAAGTTCAAGGTGTTGGG
CAACACCGACCGCCACAGTATTAAGAAGAACCTGATCGGCGCACTGTTGTTTGA
TTCCGGGGAAACGGCAGAACGCACGCGTCTTAAGCGCACGGCTCGCCGCCGC
TACACGCGTCGTAAGAATCGTATTTGCTACTTACAGGAGATTTTTTCGAATGAAA
TGGCGAAGGTTGATGATTCATTTTTCCATCGCCTTGAAGAGAGCTTTTTAGTGG
AGGAAGATAAAAAACATGAGCGTCATCCGATCTTTGGCAATATCGTAGACGAGG
TTGCCTATCACGAGAAGTACCCCACTATTTACCACCTGCGCAAGAAACTTGTCG
ACAGCACCGACAAGGCCGACCTGCGCCTGATCTATTTAGCCCTTGCGCACATG
ATCAAGTTCCGTGGTCATTTTCTGATTGAAGGCGACTTGAATCCGGACAATTCA
GACGTAGACAAGTTGTTTATTCAACTGGTTCAGACCTACAACCAATTGTTTGAG
GAAAACCCAATCAACGCATCGGGGGTGGACGCAAAGGCGATTTTATCAGCGCG
TTTGTCCAAAAGCCGCCGTCTGGAAAACCTTATTGCTCAGCTTCCAGGTGAGAA
GAAGAACGGACTTTTCGGGAACCTTATTGCGCTGAGTCTTGGTCTGACTCCGAA
TTTCAAGTCCAATTTCGACTTAGCCGAGGATGCAAAGTTACAATTGAGTAAGGA
CACGTACGATGATGACTTAGATAATCTGTTGGCGCAAATCGGTGACCAATACGC
TGACCTTTTTCTTGCAGCCAAAAACCTGAGTGACGCGATCCTGCTTAGCGATAT
CTTACGCGTGAACACTGAGATCACAAAAGCACCTCTGAGCGCCTCGATGATCAA
GCGTTATGATGAGCATCACCAGGACCTTACCTTGCTTAAAGCTCTGGTGCGCCA
GCAATTACCTGAAAAATACAAAGAGATCTTTTTTGACCAAAGCAAAAACGGTTAC
GCCGGGTATATTGATGGAGGCGCAAGCCAGGAGGAATTCTACAAGTTCATTAA
ACCTATTTTAGAAAAAATGGATGGCACGGAAGAATTGTTGGTGAAATTAAATCGT
GAAGATTTATTGCGCAAACAACGCACCTTTGATAACGGGTCGATCCCGCACCAG
ATTCATTTAGGGGAACTGCATGCTATCTTGCGTCGTCAAGAGGATTTTTACCCC
TTTTTGAAAGATAATCGCGAAAAGATCGAGAAAATCTTGACGTTTCGCATCCCAT
ATTATGTGGGCCCTTTAGCACGCGGTAATTCGCGTTTTGCATGGATGACACGTA
AGTCAGAAGAGACTATTACCCCGTGGAATTTCGAAGAAGTCGTAGACAAGGGT
GCATCCGCCCAATCATTCATTGAGCGTATGACAGCTTTTGACAAGAATTTGCCT
AACGAAAAGGTACTGCCTAAGCACTCACTGTTGTACGAGTACTTCACCGTTTAT
AATGAGCTGACAAAGGTGAAGTACGTTACTGAAGGCATGCGTAAGCCTGCCTT
CCTTTCCGGAGAACAAAAAAAAGCGATTGTAGACCTGTTATTTAAAACTAATCGT
AAAGTTACTGTTAAACAACTTAAAGAGGATTATTTTAAGAAAATTGAATGCTTTGA
TAGTGTAGAGATCAGTGGCGTAGAGGATCGCTTTAATGCGAGTCTGGGGACCT
ACCATGACCTGCTGAAGATCATTAAGGATAAAGATTTTCTGGACAATGAAGAAA
ATGAGGACATCTTAGAAGACATCGTGTTAACGCTGACGTTGTTCGAAGATCGTG
AAATGATTGAAGAGCGTCTTAAAACATACGCGCATTTGTTCGATGATAAGGTGA
TGAAGCAGTTGAAGCGTCGTCGTTACACTGGATGGGGAGCTCTGTCTCGCAAG

CTTATTAATGGTATTCGCGATAAGCAGAGCGGGAAGACCATTCTGGATTTCCTG
AAGTCTGACGGATTCGCCAATCGCAACTTCATGGCTCTTATTCACGATGATAGC
TTGACATTTAAAGAGGATATTCAGAAGGCCCAAGTATCTGGGCAAGGTGACTCC
CTTCATGAACATATCGCCAACTTGGCGGGATCCCCGGCCATCAAGAAGGGAAT
CTTACAAACAGTCAAAGTCGTGGACGAGCTGGTAAAGGTGATGGGTCGCCATA
AACCAGAAAATATCGTGATCGAAATGGCACGTGAGAATCAAACCACGCAAAAAG
GCCAAAAAAATTCTCGCGAACGTATGAAGCGCATCGAAGAGGGTATTAAGGAG
CTGGGCTCGCAAATTTTGAAGGAGCATCCAGTAGAAAATACTCAGTTACAGAAC
GAGAAGCTTTATTTGTATTATCTTCAAAACGGCCGCGACATGTATGTGGATCAA
GAGTTAGACATCAATCGTCTTAGTGACTACGATGTTGATGCTATTGTGCCACAG
AGCTTCTTGAAGGACGATAGTATCGACAACAAAGTCCTTACTCGCTCCGACAAG
AACCGCGGAAAAAGTGACAATGTTCCCAGTGAAGAAGTAGTTAAGAAGATGAAG
AACTACTGGCGTCAATTACTTAATGCCAAACTTATCACCCAGCGCAAATTCGATA
ATCTTACCAAGGCCGAACGCGGCGGTCTTTCGGAGTTGGACAAAGCAGGGTTC
ATTAAGCGCCAGCTGGTGGAAACGCGCGCGATTACAAAGCACGTCGCACAGAT
TTTAGATAGTCGTATGAATACAAAATATGACGAGAATGATAAACTGATCCGCGAA
GTAAAAGTAATTACCCTTAAGAGCAAGTTAGTGAGTGATTTTCGTAAAGATTTTC
AGTTTTATAAGGTTCGCGAGATCAATAATTACCACCACGCTCATGATGCTTATTT
AAACGCCGTCGTAGGTACCGCACTTATCAAGAAATATCCCAAACTTGAGTCTGA
ATTTGTATACGGTGATTATAAAGTGTACGACGTTCGCAAGATGATTGCTAAGAG
CGAGCAAGAGATTGGGAAAGCTACCGCCAAATACTTCTTTTATTCTAATATCATG
AATTTCTTTAAGACTGAAATTACATTGGCAAATGGCGAGATTCGCAAGCGTCCG
TTAATCGAAACTAATGGTGAGACCGGTGAAATCGTGTGGGACAAGGGCCGCGA
TTTTGCTACCGTACGCAAGGTCTTATCGATGCCTCAAGTTAACATTGTCAAGAA
GACCGAGGTTCAAACCGGTGGGTTCTCGAAGGAATCTATTCGTCCCAAACGTA
ACTCCGACAAGTTAATTGCGCGCAAAAAGGATTGGGATCCAAAGAAATATGGCG
GCTTCTTATGGCCAACCGTTGCATACTCGGTCCTTGTCGTAGCGAAGGTTGAGA
AAGGCAAGTCCAAAAAGCTTAAGTCAGTGAAGGAGTTATTAGGTATCACTATCA
TGGAACGCTCATCGTTCGAAAAGAATCCTATTGACTTCTTGGAAGCTAAAGGCT
ATAAAGAAGTAAAAAAGGACTTAATCATTAAGCTGCCAAAGTATAGTTTATTTGA
ATTGGAGAACGGACGCAAGCGTATGCTTGCCTCGGCAAAACAGTTACAGAAAG
GCAACGAATTGGCTCTTCCGAGCAAATACGTCAACTTTCTGTATCTGGCATCAC
ATTATGAGAAACTTAAAGGTTCTCCGGAAGACAACGAGCAGAAACAATTATTCG
TGGAGCAACATAAACACTATTTAGATGAAATCATTGAGCAGATCTCTGAATTTAG
TAAACGTGTCATTCTGGCTGACGCAAATTTGGATAAAGTTTTGTCAGCGTACAAT
AAACATCGCGACAAACCTATTCGCGAGCAGGCGGAAAACATTATCCATTTATTT
ACGTTGACACGCTTAGGGGCCCCCCGTGCGTTTAAGTATTTTGACACCACCATT
GATCCTAAACAGTATCGTAGCACCAAAGAAGTCCTTGATGCTACCTTAATCCAT

CAATCCATCACGGGTTTGTACGAGACACGCATCGATTTATCCCAGTTGGGCGG
CGAT

Amino acid sequence of dSpRY:

MDKKYSIGLAIGTNSVGWAVITDEYKVPSKKFKVLGNTDRHSIKKNLIGALLFDSGET
AERTRLKRTARRRYTRRKNRICYLQEIFSNEMAKVDDSFFHRLEESFLVEEDKKHE
RHPIFGNIVDEVAYHEKYPTIYHLRKKLVDSTDKADLRLIYLALAHMIKFRGHFLIEGD
LNPDNSDVDKLFIQLVQTYNQLFEENPINASGVDAKAILSARLSKSRRLENLIAQLPG
EKKNGLFGNLIALSLGLTPNFKSNFDLAEDAKLQLSKDTYDDDLDNLLAQIGDQYAD
LFLAAKNLSDAILLSDILRVNTEITKAPLSASMIKRYDEHHQDLTLLKALVRQQLPEKY
KEIFFDQSKNGYAGYIDGGASQEEFYKFIKPILEKMDGTEELLVKLNREDLLRKQRT
FDNGSIPHQIHLGELHAILRRQEDFYPFLKDNREKIEKILTFRIPYYVGPLARGNSRFA
WMTRKSEETITPWNFEEVVDKGASAQSFIERMTAFDKNLPNEKVLPKHSLLYEYFT
VYNELTKVKYVTEGMRKPAFLSGEQKKAIVDLLFKTNRKVTVKQLKEDYFKKIECFD
SVEISGVEDRFNASLGTYHDLLKIIKDKDFLDNEENEDILEDIVLTLTLFEDREMIEERL
KTYAHLFDDKVMKQLKRRRYTGWGALSRKLINGIRDKQSGKTILDFLKSDGFANRN
FMALIHDDSLTFKEDIQKAQVSGQGDSLHEHIANLAGSPAIKKGILQTVKVVDELVKV
MGRHKPENIVIEMARENQTTQKGQKNSRERMKRIEEGIKELGSQILKEHPVENTQL
QNEKLYLYYLQNGRDMYVDQELDINRLSDYDVDAIVPQSFLKDDSIDNKVLTRSDK
NRGKSDNVPSEEVVKKMKNYWRQLLNAKLITQRKFDNLTKAERGGLSELDKAGFIK
RQLVETRAITKHVAQILDSRMNTKYDENDKLIREVKVITLKSKLVSDFRKDFQFYKVR
EINNYHHAHDAYLNAVVGTALIKKYPKLESEFVYGDYKVYDVRKMIAKSEQEIGKAT
AKYFFYSNIMNFFKTEITLANGEIRKRPLIETNGETGEIVWDKGRDFATVRKVLSMPQ
VNIVKKTEVQTGGFSKESIRPKRNSDKLIARKKDWDPKKYGGFLWPTVAYSVLVVA
KVEKGKSKKLKSVKELLGITIMERSSFEKNPIDFLEAKGYKEVKKDLIIKLPKYSLFEL
ENGRKRMLASAKQLQKGNELALPSKYVNFLYLASHYEKLKGSPEDNEQKQLFVEQ
HKHYLDEIIEQISEFSKRVILADANLDKVLSAYNKHRDKPIREQAENIIHLFTLTRLGAP
RAFKYFDTTIDPKQYRSTKEVLDATLIHQSITGLYETRIDLSQLGGD
